# A new method for the joint estimation of instantaneous reproductive number and serial interval during epidemics

**DOI:** 10.1371/journal.pcbi.1011021

**Published:** 2023-03-31

**Authors:** Chenxi Dai, Dongsheng Zhou, Bo Gao, Kaifa Wang

**Affiliations:** 1 State Key Laboratory of Pathogen and Biosecurity, Beijing Institute of Microbiology and Epidemiology, Beijing, China; 2 School of Mathematics and Statistics, Southwest University, Chongqing, China; The University of Hong Kong, CHINA

## Abstract

Although some methods for estimating the instantaneous reproductive number during epidemics have been developed, the existing frameworks usually require information on the distribution of the serial interval and/or additional contact tracing data. However, in the case of outbreaks of emerging infectious diseases with an unknown natural history or undetermined characteristics, the serial interval and/or contact tracing data are often not available, resulting in inaccurate estimates for this quantity. In the present study, a new framework was specifically designed for joint estimates of the instantaneous reproductive number and serial interval. Concretely, a likelihood function for the two quantities was first introduced. Then, the instantaneous reproductive number and the serial interval were modeled parametrically as a function of time using the interpolation method and a known traditional distribution, respectively. Using the Bayesian information criterion and the Markov Chain Monte Carlo method, we ultimately obtained their estimates and distribution. The simulation study revealed that our estimates of the two quantities were consistent with the ground truth. Seven data sets of historical epidemics were considered and further verified the robust performance of our method. Therefore, to some extent, even if we know only the daily incidence, our method can accurately estimate the instantaneous reproductive number and serial interval to provide crucial information for policymakers to design appropriate prevention and control interventions during epidemics.

## Author summary

Contagious disease epidemics, such as coronavirus disease 2019 (COVID-19), intermittently threaten global public health. In this situation, policymakers usually hope to timely monitor the severity of the disease transmission and thus evaluate the effectiveness of the prevention and control interventions. Instantaneous reproductive number and serial interval are the two most commonly used monitoring indices. The former refers to the expected number of secondary cases infected by a primary case, and the latter refers to the time interval between a primary case presenting with symptoms and its secondary cases developing symptoms. However, the existing frameworks can hardly estimate these two indicators simultaneously in the absence of additional contact tracing data or other prior information. In the presented study, we developed a likelihood method incorporating interpolation, Bayesian information criterion and Markov Chain Monte Carlo method to jointly estimate the two quantities, which only depends on the daily incidence. The simulation study and several data sets of historical epidemics verified the robust performance of our method. Therefore, even in the case of outbreaks of emerging infectious diseases with an unknown natural history or undetermined characteristics, our method can, to a certain extent, provide crucial information for policymakers to design appropriate measures during epidemics.

## 1. Introduction

Contagious disease epidemics, such as the Spanish flu, smallpox, severe acute respiratory syndrome (SARS), and coronavirus disease 2019 (COVID-19), intermittently threaten global public health [1–4]. Currently, COVID-19 has spread worldwide since its initial outbreak in 2019, leading to an ongoing pandemic [5]. Mathematical models have been important tools to help policymakers design appropriate prevention and control interventions [6–8]. In these models, the serial interval and the reproductive number are regarded as the two most important parameters for understanding disease transmission [9].

The serial interval is defined as the time interval between a primary case presenting with symptoms and its secondary cases developing symptoms [10]. It is a mixture of the incubation period and infectious period, both of which are easy to understand but difficult to measure. Therefore, this quantity can only be estimated through detailed, time-consuming and expensive contact tracing.

The time-varying reproductive number is widely used to assess whether a disease will continue to spread under current control efforts [11], and its value denotes the expected number of secondary cases arising from a primary case infected at time *t*. In general, if the value is less than one, the outbreak will die out, while if it is greater than one, a sustained outbreak is likely. Thus, control measures must be implemented to decrease the time-varying reproductive number in practice.

Two formal definitions of the time-varying reproductive number have been proposed: case reproductive number $R_{t}^{c}$ and instantaneous reproductive number *R*_*t*_ [12]. By considering all possible transmission trees consistent with the observed epidemic data, the case reproductive number is estimated at each timestep with observed cases [8]. Due to its definition, this parameter can be estimated only in a delayed manner. For instance, if a policymaker wishes to understand the effects of control interventions in real time, this parameter may not be useful because it does not change immediately after interventions are altered [13]. In contrast, the instantaneous reproductive number changes immediately without delay and is therefore a useful quantity for understanding the effects of control strategies in real time [6]. Recognizing this advantage, Cori et al. subsequently developed a method and software (the EpiEstim R package) to estimate the instantaneous reproductive number with two inputs: daily incidence and the distribution of the serial interval [6]. Thompson et al. extended this statistical framework by integrating data on known pairs of primary cases and secondary cases from which the serial interval is directly estimated rather than relying on a priori estimates of the serial interval [13]. Although these methods [6,13] have been widely used, they may be limited in some contexts because of an important limitation, i.e., the serial interval and instantaneous reproductive number cannot be simultaneously estimated based on the daily incidence alone, resulting in the need for additional contact tracing data on the serial interval, which is often difficult and expensive to attain. In fact, in the initial phase of an outbreak, the serial interval is often not available or may be associated with significant uncertainty. This limitation is particularly notable for outbreaks of emerging infectious diseases with an unknown natural history or undetermined characteristics, as well as outbreaks of a known infectious diseases in a new population [14]. Although White et al. introduced a method to estimate both quantities and applied it to data from the 1918 influenza outbreak in a previous study [1], the approach is suitable only for a two-phase epidemic (an outbreak followed by control interventions). Thus, an improved method is required for complicated epidemic curves, such as two-wave epidemics and/or seasonal epidemics.

In this study, we extend the statistical framework [7] for estimating the instantaneous reproductive number and the serial interval directly from daily incidence without additional contact tracing data. The paper is organized as follows. After describing our method, we apply it first to simulated data from two artificial scenarios and then to several historical epidemics, including pandemic influenza [1], smallpox [2], SARS [3], COVID-19 [15,16], and a seasonal epidemic, hand-foot-mouth disease (HFMD) [17].

## 2. Materials and methods

### 2.1. Statistical method

**Fig 1:**
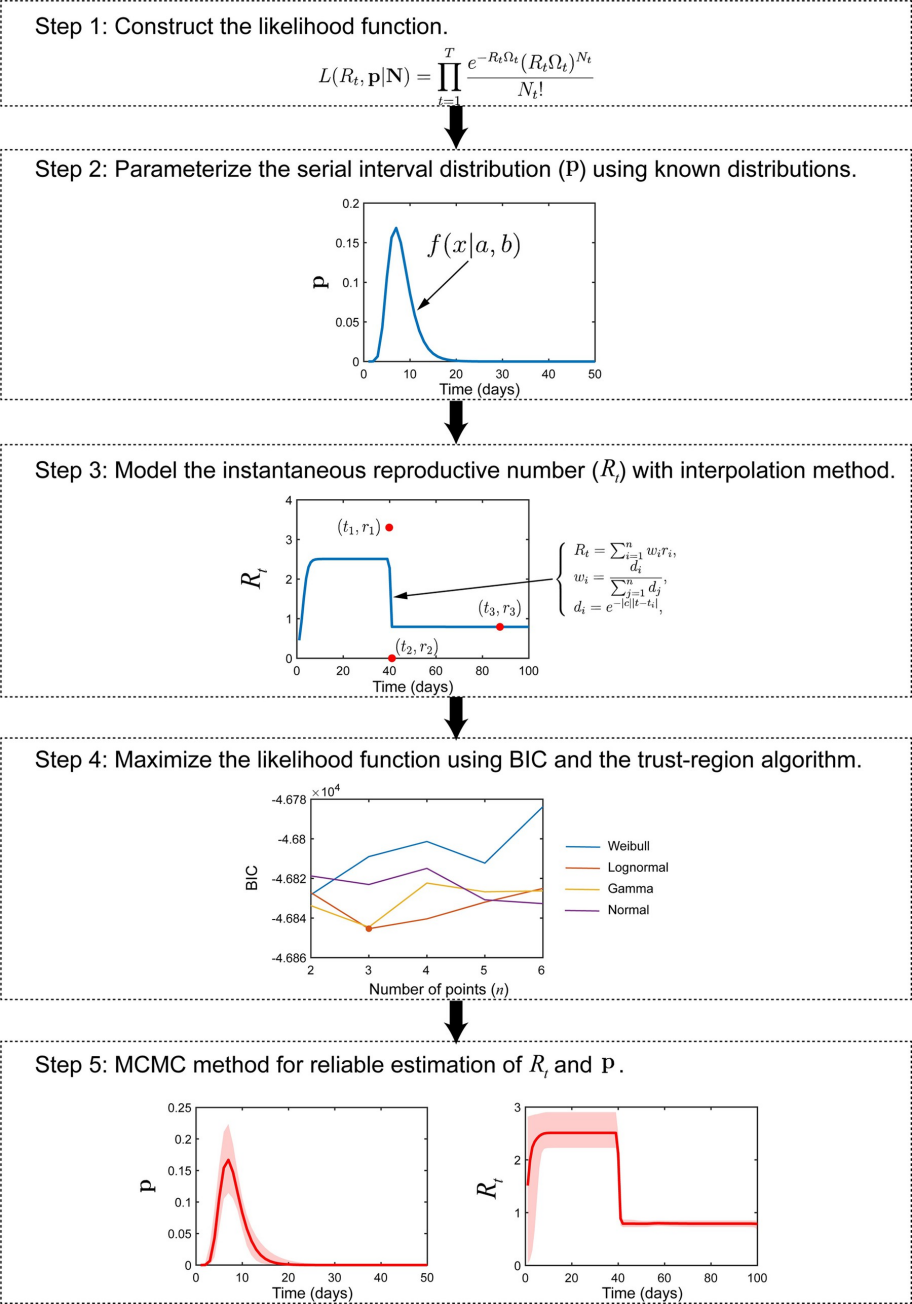
A precise illustration of the framework for estimating the serial interval and the instantaneous reproductive number using daily incidence time series. Step 1: Construct the likelihood function of the instantaneous reproductive number and the serial interval. Step 2: Parameterize the serial interval by known distributions. Step 3: Model the instantaneous reproductive number parametrically as a function of time using the interpolation method. Step 4: Employ the BIC and trust-region algorithm to maximize the likelihood function and tune undetermined parameters. Step 5: Use the MCMC method to estimate the target distribution of the instantaneous reproductive number and the serial interval. BIC: Bayesian information criterion; MCMC: Markov Chain Monte Carlo.

Inspired by a previous study [7] (see S1 Text for details), we proposed an improved likelihood-based methodology to jointly estimate the instantaneous reproductive number and the serial interval using only daily incidence. The proposed method is divided into five key steps (Fig 1), and the specific contents of each step are described below. Firstly, we constructed the likelihood function of the instantaneous reproductive number and the serial interval. Secondly, the serial interval was parameterized by known distributions. Thirdly, the instantaneous reproductive number was modeled parametrically as a function of time using the interpolation method; Fourthly, the Bayesian information criterion (BIC) and the trust-region algorithm were employed to maximize the likelihood function and tune the undetermined parameters; Finally, the robust adaptive Metropolis (RAM) algorithm [18] was used to conduct the Markov Chain Monte Carlo (MCMC) procedure and estimate the target distribution of the instantaneous reproductive number as well as the serial interval. Notably, data obtained from Steps 4 and 5 can both be used as the final results. The MCMC method (MATLAB toolbox) was further utilized to enhance the credibility, and obtain the distribution and 95% credible interval (95% CI) of all parameters. Thus, data from Step 4 are usually reported as the final results for simulation study to save computational cost, while data from Step 5 for historical epidemic data sets.

#### 2.1.1. Likelihood function of the instantaneous reproductive number and the serial interval

Let ***N*** = {*N*_*t*_}, *t* = 0,1,2⋯*T* represent the number of new cases per day between days 0 and *T* of the epidemic, which is the unique known data set in the procedure, and let *X*_*ij*_ represent the number of cases that appear on day *j* that are infected by individuals who develop symptoms on day *i*, which usually represents hidden, unknown data. An illustration of the model for disease transmission in the population is provided below.

$$\begin{array}{l} N_{0}\\ N_{1}=X_{01}\\ N_{2}=X_{02}+X_{12}\\ N_{3}=X_{03}+X_{13}+X_{23}\\ N_{4}=X_{04}+X_{14}+X_{24}+X_{34}\\ \;\vdots =\vdots \\ N_{T}=\sum_{i=T-\min(T,k)}^{T-1}X_{iT} \end{array}$$

in which *k* denotes the length of the serial interval.

Similar to a previous report [7], we assumed that the total number of cases produced by *N*_*t*_ on day *t*, $X_{t,.}=\sum_{i=1}^{k}X_{t,t+i}$, exhibited a Poisson distribution with parameter *N*_*t*_*R*_*t*_, where *R*_*t*_ is the reproductive number for cases on day *t*. Furthermore, $X_{t}=\{X_{t,t+1},X_{t,t+2},\cdots ,X_{t,t+k}\}$ was assumed to follow a multinomial distribution $\left(X_{t}∼PN(X_{t,.},p)\right)$ with parameters (*X*_*t*,._, **p**, *k*), where **p** = {*p*_1_, *p*_2_,⋯,*p*_*k*_} represents the distribution of the serial interval. According to the aforementioned assumptions and several pioneering works [6,7], we constructed a likelihood function (see S1 Text for details). After simplification, it yields the following convenient form:

$$L(R_{t},p|N)=\prod_{\tau =1}^{t}\frac{e^{-R_{\tau }\Omega_{\tau }}(R_{\tau }\Omega_{\tau }{)}^{N_{\tau }}}{N_{\tau }!},$$ (1)

where $\Omega_{t}=\sum_{i=1}^{\min(k,t)}N_{t-i}p_{i}$. Maximization of the likelihood function (1) yields estimates of *R*_*t*_ and **p**.

#### 2.1.2. Parameterized serial interval distribution by known distributions

Serial interval instead of generation time was estimated, because the latter is often difficult to acquire in practice and its distribution is difficult to validate. Notably, Weibull, lognormal, gamma, and normal distributions (Table 1) are frequently used to fit serial interval distributions of various diseases [19,20], which are two-parameter distributions that provide rich family with sufficient flexibility to model a large number of infectious disease data sets. The criterion for choosing a distribution is whether it yields the largest value of likelihood function (1) among the four distributions. To reduce the dimensionality of the parameter space and create a concise and reliable model, **p** were parameterized by these distributions. Therefore, we mimicked *p*_*i*_ as

$$\left\{ \begin{array}{l} \psi_{i}=\int_{i-1}^{i}f(x|a,b)dx,\\ p_{i}=\frac{\psi_{i}}{\sum_{j=1}^{k}\psi_{j}},i=1,2,\cdots ,k, \end{array}\right.$$ (2)

where *f*(*x*|*a*, *b*) is the probability density function of the serial interval. This formulation means that we discretized the distribution and, since *k* is finite, truncate it as well. *ψ*_*i*_ was normalized to ensure that the sum is unit so that **p** can represent a probability distribution. Since *k* was reported to exert a trivial impact on the results if *k* is sufficiently large [7], we specified the formula with the constraint that *k*<*T*. Furthermore, the normal distribution we used in this study was a truncated form, since our model assumed that only patients on day *t* would be infected by individuals who developed symptoms before day *t*. We set *a*>0, *b*>0 for Weibull, gamma, and truncated normal distributions, and *a*∈ℝ, *b*>0 for lognormal distribution to ensure the positive value of serial interval.

**Table 1:** Distributions used to estimate the serial interval.

Distribution	Probability density function	Parameters
Weibull	$f(x|a,b)=ab(ax{)}^{b-1}e^{-(ax{)}^{b}},x\geq 0$	*a*>0, *b*>0
Lognormal	$f(x|a,b)=\frac{b}{\sqrt{2\pi }x}e^{-2b^{2}(\ln(x)-a{)}^{2}},x>0$	*a*∈ℝ, *b*>0
Gamma	$f(x|a,b)=\frac{b^{a}}{\Gamma (a)}x^{a-1}e^{-bx},x\geq 0$	*a*>0, *b*>0
Truncated normal	$f(x|a,b)=\frac{1}{\sqrt{2\pi b^{2}}}e^{-\frac{(x-a{)}^{2}}{2b^{2}}},x\geq 0$	*a*>0, *b*>0

#### 2.1.3. The instantaneous reproductive number was modeled parametrically as a function of time using the interpolation method

Similarly, *R*_*t*_ was modeled parametrically as a function of time using an interpolation method with some data points {*t*_*j*_, *r*_*j*_}, *j* = 1,2,⋯,*n* (Fig 1, Step 3, red points), where *n* is the number of data points used for the interpolation of *R*_*t*_. The data points {*t*_*j*_, *r*_*j*_} are key variables because they determine the shape of *R*_*t*_ and the value of the likelihood function. Although numerous interpolation methods were available, including linear interpolation, polynomial interpolation, spline interpolation, and nearest-neighbor interpolation [1,21], these methods required {*t*_*j*_}, *j* = 1,2,⋯,*n* in monotonous order, and it was not easy to extrapolate when *t*<*t*_1_ or *t*>*t*_*n*_. Here, we provided an interpolation method without the aforementioned limitations, called the adaptive weighted neighbors method (AWN). Similar to Gaussian process/Kriging, AWN is also a kernel-based method that uses a limited set of sampled data points to estimate the value of a variable over a continuous spatial field (see our previous study for details [22]). Specifically, *R*_*t*_ was estimated using the following formulas:

$$\left\{ \begin{array}{l} R_{t}=\sum_{i=1}^{n}w_{i}r_{i},\\ w_{i}=\frac{d_{i}}{\sum_{j=1}^{n}d_{j}},\\ d_{i}=e^{-|c||t-t_{i}|}, \end{array}\right.$$ (3)

where *t*_*i*_≥0, *r*_*i*_≥0, *n*≥2, *i* = 1,2,⋯,*n*. AWN made full use of the information contained in all key variables {*t*_*j*_, *r*_*j*_}, *j* = 1,2,⋯,*n* and automatically assigned greater weight to nearby interpolating points. The closer *t* is to *t*_*i*_, the smaller *d*_*i*_ and the greater *w*_*i*_, resulting in *R*_*t*_ closer to *r*_*i*_. The prior distribution of *r*_*j*_ is *U*(0.5,5), where *U* represents a uniform distribution. These variables {*t*_*j*_, *r*_*j*_}, *j* = 1,2,⋯,*n*, parameter *c* and the aforementioned interpolation method (3) fit any shapes of *R*_*t*_ by increasing *n* and tuning *c*, which describe the timing of the intervention and the rapidity with which it affects transmission.

#### 2.1.4. BIC and the trust-region algorithm were employed to maximize the likelihood function and tune undetermined parameters

When fitting model (1), although the likelihood *L*(*R*_*t*_, **p**|***N***) was increased by adding parameters, i.e., increasing *n*, doing so may result in overfitting and lower accuracy. According to a previous study [23], we introduced a penalty term for the number of parameters in the model and used BIC to resolve this problem. Concretely, the BIC was defined as follows:

$$BIC=-2\;\ln\;L(R_{t},p|N)+(2n+3)\;\ln\;T$$ (4)

where 2*n*+3 is the number of undetermined parameters and *T* is the number of data points of ***N***. Similar to a previous report [24], an iterative method called the trust-region algorithm was used to minimize BIC and tune the parameters by supplying the gradients of the negative log-likelihood −ln *L*(*R*_*t*_, **p**|***N***) with respect to the parameters (see S1 Text for details). According to the BIC results, we obtained the number of parameters, the number of data points (*n*), the distribution of the serial interval, and the values for all parameters, **p** and *R*_*t*_ (Fig 1, Step 4).

#### 2.1.5. The MCMC method was used to generate the distribution of the instantaneous reproductive number and the serial interval

From Subsection 2.1.4, we obtained the results of parameters as the initial values for the chain, and the parameters were constrained by $0\leq a,b,c,0\leq t_{j}\leq t,0\leq r_{j},j=1,2,\cdots ,n$ (see details in S1 Table). The MCMC method based on MATLAB toolbox requires five elements for each parameter, including initial value, minimum value, maximum value, prior mean, and standard deviation (if the parameter is assumed to be normally distributed), but only the initial value must be provided and other elements are optional. S1 Table showed the prior information for each parameter. Then the RAM algorithm [18] was used to conduct the MCMC procedure and estimate the target distribution of **p** and *R*_*t*_ (Fig 1, Step 5). The algorithm ran for 2×10^6^ iterations after a burn-in of 10^6^ iterations, with the Geweke convergence diagnostic method employed to assess the convergence. Finally, we reliably obtained the distribution and 95% CI of the parameters {*a*, *b*, *c*}, key variables {*t*_*j*_, *r*_*j*_}, *j* = 1,2,⋯,*n*, **p** and *R*_*t*_. Additionally, the codes of the proposed algorithm are available in S1 Codes.

### 2.2. Simulation study

We designed a simulation study based on two artificial scenarios to assess the ability of our proposed method to quantify the transmissibility of epidemics (see S1 Text for details). In scenario one, we verified the effectiveness of our method by changing different parameters, including the choice of Akaike information criterion (AIC) or BIC, the number of initial cases, the distribution of the serial interval, the mean and variance of the serial interval, the epidemic severity, the subsequent effectiveness of control measures, timeliness and other parameters. Then, we compared our approach with two previous methods (hereafter referred to as White et al method [1] and Cori et al method [6]). In scenario two, the outbreak-control-rebound-control epidemic curve was employed to verify that our method would fit any shape of curve for the instantaneous reproductive number and serial interval.

#### 2.2.1. Scenario one

Scenario one assumed that an epidemic had a constant *R*_*t*_ (*R*_1_>1) before a certain date (day 40) and then had another constant value (*R*_2_<1) to simulate the effect of control measures, such as school closures. We applied our method to an example in this scenario to illustrate the working principles and verify that it produced sensible results. This example assumed that the initial number of cases (*N*_0_) was 2, the serial interval was lognormal distributed with mean and variance *μ* = 8, *σ*^2^ = 9, and *R*_1_ = 2.5, *R*_2_ = 0.9. In addition, we illustrated the reason that the BIC instead of the AIC was chosen in this study.

Furthermore, the influence of different epidemic parameters (serial interval distribution, *μ*, *σ*^2^, *N*_0_, *R*_1_, and *R*_2_) on the effectiveness of the methods was simulated by changing the evaluation parameters while keeping the other parameters fixed. We first investigated the effects of the serial interval distribution (Weibull, lognormal, gamma, and normal). Then, the effect of the mean serial interval (*μ* = 6, *μ* = 8, and *μ* = 10) was studied. Next, we tested how the variance of the serial interval (*σ*^2^ = 4, *σ*^2^ = 9, and *σ*^2^ = 16) affected the results. Fourth, the impact of the number of initial cases *N*_0_ (2, 20) was investigated. Fifth, we tested how the epidemic severity (*R*_1_ = 2.5, *R*_1_ = 1.8) affected the results. Last, we quantified how *R*_2_ (0.8, 0.9) affected the inference results to mimic the effectiveness of control measures.

To assess the timeliness of our method, we also clarified the impact of time length of the data on the performance of our method by varying the time length from 20 to 100. Besides, we performed a prospective analysis to predict the transmissibility in the next 20 days.

Note that serial interval has been shown to change over time, even in short periods of time and around the initial stages of an epidemic [25]. We then illustrated the effectiveness of our method by changing the mean serial interval from 8 days at the beginning of the epidemic to 3 days at the end of the epidemic. We also compared the results to the case with a fixed mean serial interval of 8 days.

Finally, we implemented the White et al method [1] and Cori et al method [6], and compared the results of the three methodologies. White et al method implemented a logistic curve that is suitable only for two-stage epidemics, including outbreak and control stages. The likelihood for the method was fitted using a Nelder-Mead maximization procedure, and 100 starting values were used to ensure that the global maximum was reached. This method was summarized in S2 Text. Cori et al. developed a method and software (the EpiEstim R package) for estimating the instantaneous reproductive number, but the method relies on the distribution of serial intervals. Thus, we calculated the results of Cori et al method based on the ground truth of serial interval. We also summarized Cori et al method in S2 Text.

#### 2.2.2. Scenario two

Scenario two simulated four two-wave epidemics to verify whether our method would fit any shapes of *R*_*t*_. *R*_*t*_ was constant during the initial outbreak (*R*_1_, *T*_1_), control stage (*R*_2_, *T*_2_), rebound (*R*_3_, *T*_3_) and recontrol stage (*R*_4_, *T*_4_). *R*_1_−*R*_4_ and *T*_1_−*T*_4_ are two kinds of key parameters that determine the epidemic severity and duration of each stage, respectively. We assumed that *N*_0_ = 2 and the serial interval exhibited a lognormal distribution with a mean and variance of *μ* = 8 and *σ*^2^ = 9, respectively. Besides, we assessed the timeliness and performed a prospective analysis in this complicated scenario.

#### 2.2.3. Quantitative validation measures

To evaluate the performance of our method, three indicators, *ΔR*_*t*_, *Δμ*, and *Δσ*, were used in this study.

*ΔR*_*t*_ was employed to evaluate the degree of similarity between the ground truth and the estimate of the instantaneous reproductive number, which was calculated using the following Eq:

$$\Delta R_{t}=\frac{1}{T}\sum_{t=1}^{T}\left|R_{t}-{\hat{R}}_{t}\right|,$$ (5)

where *R*_*t*_ and ${\hat{R}}_{t}$ denote the ground truth and estimate, respectively. A lower *ΔR*_*t*_ represents a higher accuracy of ${\hat{R}}_{t}$.

*Δμ* and *Δσ* were used to evaluate the performance of serial interval estimates,

$$\Delta \mu =|\mu -\hat{\mu }|,$$ (6)

$$\Delta \sigma =|\sigma -\hat{\sigma }|,$$ (7)

where *μ* and *σ* are the ground truth mean and standard deviation of the serial interval, respectively, and $\hat{\mu }$ and $\hat{\sigma }$ are the corresponding estimates. Similarly, lower *Δμ* and *Δσ* represent more accurate estimates.

### 2.3. Application to historical epidemics

To illustrate that our proposed method can provide new insights, we applied it to seven historical epidemics that varied in terms of reproductive number, serial interval, and epidemic scale. We retrieved the epidemic curves, as well as the means and standard deviations of the serial interval from the literatures and systematic reviews (Table 2). Meanwhile, we compared our method with two other methods: White et al method [1] and Cori et al method [6] (see S2 Text for details).

**Table 2:** Historical epidemics and their serial interval.

Disease	Location	Year of Epidemic	Serial Interval, days (Mean±SD)		
			Reported values	Our method	White et al method
Pandemic influenza [1]	Boonah	1918	3.81±1.12 [1,28]	4.00(95% CI, 2.74–5.19) ± 1.35(95% CI, 0.82–2.40)	3.81±1.12
Pandemic influenza [1]	Cumberland	1918	8.28±5.00 [1,28]	7.14(95% CI, 5.52–8.87) ± 3.86(95% CI, 2.83–4.98)	8.28±5.00
Smallpox [2]	Kosovo	1972	22.4±6.1 [29], 9–45 [19], 18.6 [30]	18.85(95% CI, 18.08–19.83) ± 2.32(95% CI, 1.69–3.35)	12.06±13.39
SARS [3]	Hong Kong	2003	8.4±3.8 [19,31]	9.70(95% CI, 7.29–12.42) ± 4.57(95% CI, 3.11–6.47)	3.89±3.25
COVID-19 [15]	Hunan	2020	4.40±3.17 [15,32], 3.3–7.6 [33]	3.42(95% CI, 1.82–6.19) ± 1.48(95% CI, 0.51–3.13)	1.98±0.84
COVID-19 [16]	Chongqing	2020	5.2±6.2 [26], 3.3–7.6 [33]	3.79(95% CI, 2.81–4.91) ± 0.86(95% CI, 0.51–1.69)	3.89±0.97
Hand-foot-mouth disease [17]	Wenzhou	2010–2011	3.7±2.6 [27], 3 [34]	3.86(95% CI, 3.13–4.72) ± 5.08(95% CI, 3.77–6.53)	2.39±2.69

A total of seven historical epidemics were collected. Firstly, data from a troop ship that embarked in the late fall of 1918 were reported. Boonah left Durban and reported the first three definitive cases of influenza on November 29. Researchers who collected the data noted that some initial cases were likely not identified. A total of 427 cases were reported in the 40 days of the epidemic. Secondly, another well-documented influenza outbreak in 1918 was considered. During the pandemic, the United States Public Health Service created special surveys of 18 localities, including Maryland, where Cumberland is one of the communities. In this community, the reported results were derived from house-to-house surveys requesting the date of onset of influenza for all infected individuals. Thirdly, four other data sets, including the smallpox outbreak in Kosovo in 1972, the SARS outbreak in Hong Kong in 2003, COVID-19 in Hunan, COVID-19 in Chongqing, were analyzed. Lastly, to illustrate the effectiveness of our method for complicated epidemic curves, we adopted HFMD data in Wenzhou, mainland China, which has a distinct seasonal pattern with more than two annual peaks in subtropical regions.

### 2.4. Statistical analysis

The statistical analysis was performed with SPSS 22.0 software (SPSS, Chicago, IL, USA). Since the data for *ΔR*_*t*_, *Δμ*, and *Δσ* were not normally distributed, the Kruskal-Wallis test and its post hoc comparisons, and Wilcoxon test were used to analyze the effects of epidemic parameters (serial interval distribution, *μ*, *σ*^2^, *N*_0_, *R*_1_, *R*_2_, fixed and varying serial intervals), and AIC or BIC depending on the number of assigned groups. Friedman’s analysis of variance (ANOVA) with its post hoc test, and paired samples Wilcoxon test were used to analyze the effects of different methods (White et al, Cori et al and our methods), different time length, depending on the number of assigned groups, since their results were generated from the same data set. A two-tailed Student t-test was used for single comparison of standard deviation of serial interval between White et al and our methods). The null hypothesis of aforementioned tests stated that no differences were observed among/between groups. Chi-square test was used to measure the difference of the number of data points for *R*_*t*_ interpolation between AIC and BIC. Pearson correlation analysis was performed among parameters after MCMC analysis. P<0.05 was considered statistically significant.

## 3. Results

### 3.1. Simulated data set in scenario one

We applied our method to estimate the serial interval and instantaneous reproductive number throughout scenario one in the simulation study, for which only the daily incidence was available.

**Fig 2:**
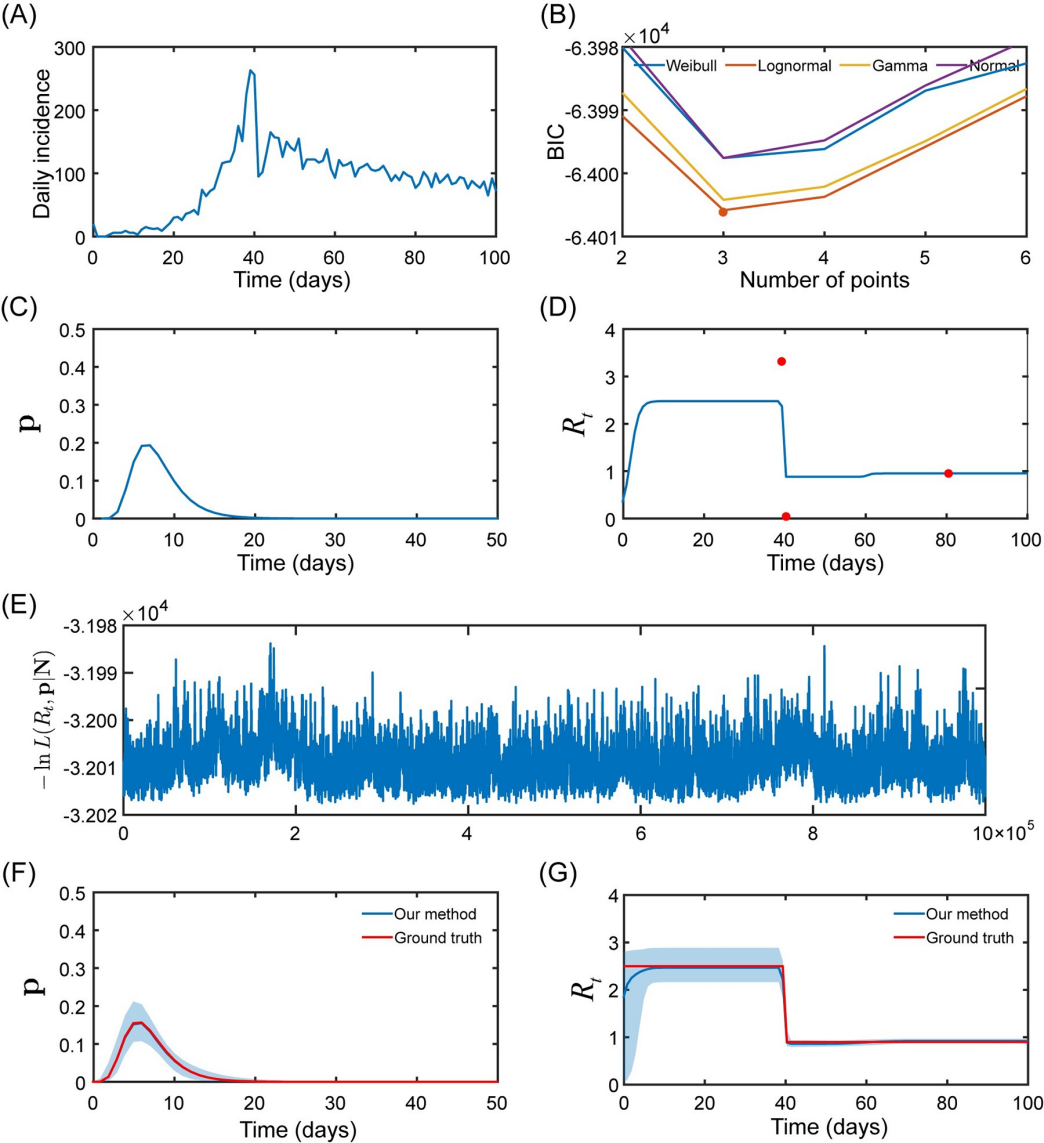
An example illustrating the workflow of our method. The time series of daily incidence (A) was simulated according to the conditions of scenario one. Using the BIC (B), the number of data points and serial interval distribution were determined, and the serial interval (C) and instantaneous reproductive number (D) were obtained. The MCMC method (E) was then used to generate the distribution of the serial interval (F) and instantaneous reproductive number (G). BIC: Bayesian information criterion; MCMC: Markov Chain Monte Carlo.

An example illustrating the workflow of our method is depicted in Fig 2. The time series of daily incidence was first generated under the condition of scenario one (*N*_0_ = 2, the serial interval obeys a lognormal distribution with *μ* = 8, *σ*^2^ = 9, and *R*_1_ = 2.5, *R*_2_ = 0.9). The daily incidence indicated that the epidemic first expanded rapidly during the outbreak and retracted gradually after a control measure on day 40. After our method was applied, the lowest value for BIC was observed when *n* = 3 and the lognormal distribution was used (Fig 2B), and the corresponding results for **p** and *R*_*t*_ were also acquired (Fig 2C and 2D). Then, the RAM algorithm was implemented to conduct the MCMC procedure (Fig 2E) and estimate the target distributions of **p** and *R*_*t*_ (Fig 2F and 2G). The MCMC chains, distributions, and correlations of 9 (2*n*+3) parameters are shown in S1 Fig. Besides, we chose the BIC since the better performance was observed when compared to AIC, such as lower *ΔR*_*t*_, *Δμ*, *Δσ*, and number of data points for *ΔR*_*t*_ interpolation (see details in S2 Fig).

To better characterize our method’s performance, we tested how six epidemic parameters affected the results (S3 Fig). The simulations were performed based on the assumptions that the number of initial cases was 2, the serial interval exhibited a lognormal distribution with a mean and variance of 8 and 9, respectively and with a constant R before (*R*_1_ = 2.5) and after (*R*_2_ = 0.9) a control measure was implemented on day 40. In addition to the use of *ΔR*_*t*_, *Δμ* and *Δσ* to assess the bias in estimates, the proportion of the 95% CI covering the true value is also important when evaluating the performance. However, we did not consider this indicator, because our method yielded high performance (0.99 [0.96 to 1.00, 95% CI]) and no difference would be detected when investigating the effects of different epidemic parameters. When we varied a parameter to study its effects, 100 epidemics were produced, and all other settings were kept unchanged. Since the distributions of the serial interval and instantaneous reproductive number are not needed when testing the effects of different parameters, we did not execute the MCMC method (Step 5). We found that the serial interval distribution exerted trivial effects on *ΔR*_*t*_, *Δμ* and *Δσ* (S3A Fig). The mean serial interval exerted a marked impact on performance; it increased consistently as *ΔR*_*t*_, *Δμ* and *Δσ* increase (S3B Fig). In addition, the variance in the serial interval was reported to increase with increasing and *Δμ*, *Δσ* except for *ΔR*_*t*_ (S3C Fig). The number of initial cases and epidemic severity also produced notable effects: larger *N*_0_ and *R*_1_ tended to yield accurate results, i.e., lower *ΔR*_*t*_, *Δμ* and *Δσ* values (S3D and S3E Fig). However, we did not observe significant effects of *R*_2_ on *ΔR*_*t*_, *Δμ* and *Δσ* (S3F Fig).

**Fig 3:**
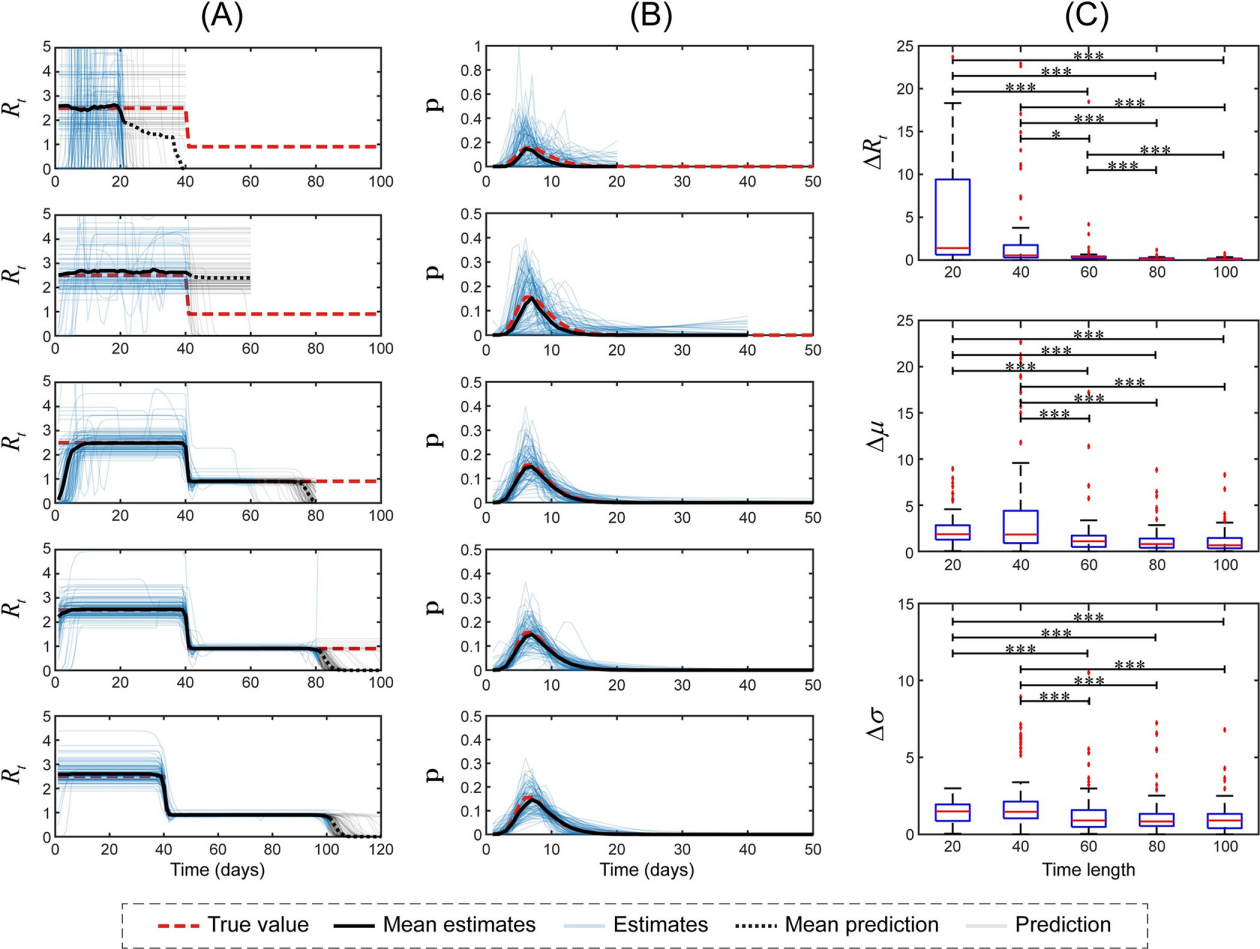
The effects of the time length on the results. A total of 100 trials were conducted to investigate the performance of our method by varying time length (from 20 to 100). Simulations were performed based on the assumptions that the number of initial cases was 2, the serial interval exhibited a lognormal distribution with a mean and variance of 8 and 9, respectively, and a constant R before (*R*_1_ = 2.5) and after (*R*_2_ = 0.9) a control measure on day 40. The left, middle and right panels are the estimates of instantaneous reproductive number, the estimates of the serial interval and the results of three indices, respectively. The blue lines show the estimates, and the dark lines denote their mean values under 100 simulations. Additionally, the grey lines denote predictions and dashed dark lines represent their mean values from 100 simulations. The red dashed lines represent the ground truth. *: P<0.05, and ***: P<0.001.

To clarify the impact of time length of the data on the performance of our method, we analyzed the same simulated data sets by varying time length from 20 to 100 (Fig 3). Obviously, a longer time length yielded better performance, such as lower *ΔR*_*t*_, *Δμ* and *Δσ*. In particular, the longer time length groups (60, 80, 100) exhibited significantly better performance than the shorter time length groups (20 and 40), including lower *ΔR*_*t*_, *Δμ* and *Δσ* values. No difference was observed among longer time length groups and between shorter length groups, except that a remarkably lower *ΔR*_*t*_ was observed for time length 80, 100 compared to that of time length 60. Besides, we found that if *R*_*t*_ remained unchanged in the next 20 days, our method could accurately predict its trend (the third row in Fig 3A); otherwise, our method would lose its accuracy (the second row in Fig 3A).

We analyzed another simulated data set to show the effectiveness of our method when the serial interval changed over time (S4 Fig). The mean serial interval changed from 8 days at the beginning of the epidemic to 3 days at the end of the epidemic (S4A and S4B Fig). The true serial interval distribution that was averaged over time (red dashed curve) was compared with our estimates in S4D Fig. Surprisingly, our method also accurately estimated *R*_*t*_ and serial interval (S4C and S4D Fig), since no differences were observed between the *ΔR*_*t*_, *Δμ* and *Δσ* for varying and fixed serial intervals (S4E, S4F and S4G Fig).

**Fig 4:**
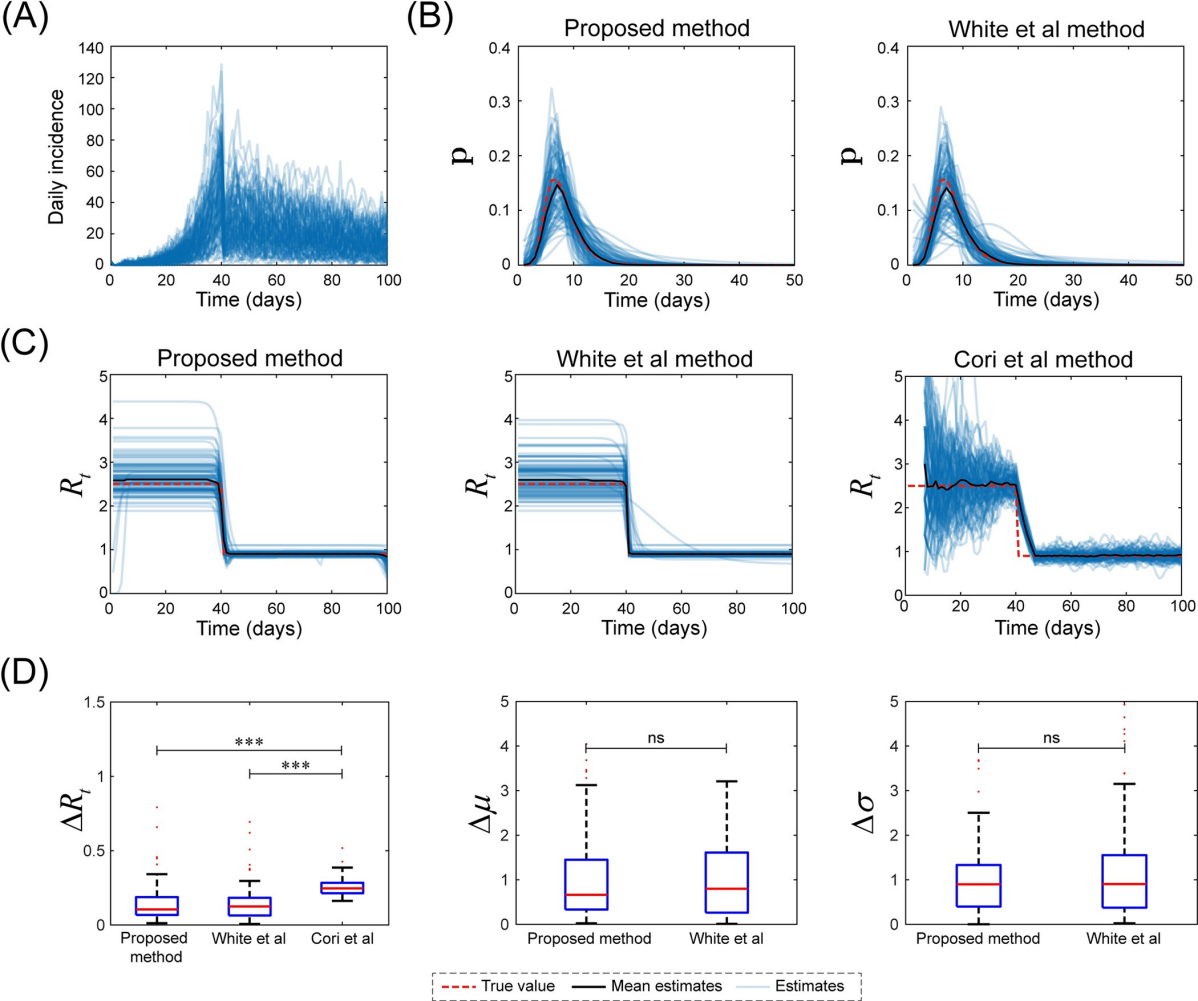
Comparison of methods in scenario one. Daily incidence (A) was obtained based on the assumptions that the number of initial cases was 2, the serial interval exhibited a lognormal distribution with a mean and variance of 8 and 9, respectively, and a constant R before (*R*_1_ = 2.5) and after (*R*_2_ = 0.9) a control measure on day 40. The results of serial interval obtained by our method and White et al method (B). The results of instantaneous reproductive number obtained by all methods (C). The red dashed lines show the ground truth. *ΔR*_*t*_, *Δμ*, and *Δσ* (D). The blue lines show the estimates, and the dark lines denote the mean values for 100 simulations. ns: no significant difference, ***: P<0.001.

We analyzed the same simulated data sets to demonstrate our method’s performance relative to that of the White et al method and Cori et al method. We included the White et al method in the performance comparisons because it was designed for the outbreak-control mode with a two-stage function. Cori et al method was employed to demonstrate the robustness of our method even in the absence of serial interval. Since the distributions of the serial interval and instantaneous reproductive number were not needed in the comparison with the White et al method and to reduce computational cost, we did not execute the MCMC method (Step 5). As expected, all methods detected changes in the instantaneous reproductive number, for instance, a decrease in transmissibility following a control measure, and precisely estimate the serial interval distribution (Fig 4C). However, we also observed the inaccurate results of Cori et al method when *R*_*t*_ changed substantially. Moreover, our method performed comparably to White et al method since no differences were observed in *ΔR*_*t*_, *Δμ* and *Δσ* (Fig 4D). We also observed better performance of our method and White et al method over Cori et al method, because of the remarkably lower *ΔR*_*t*_ of the former two methods (Fig 4D).

### 3.2. Simulated data set in scenario two

**Fig 5:**
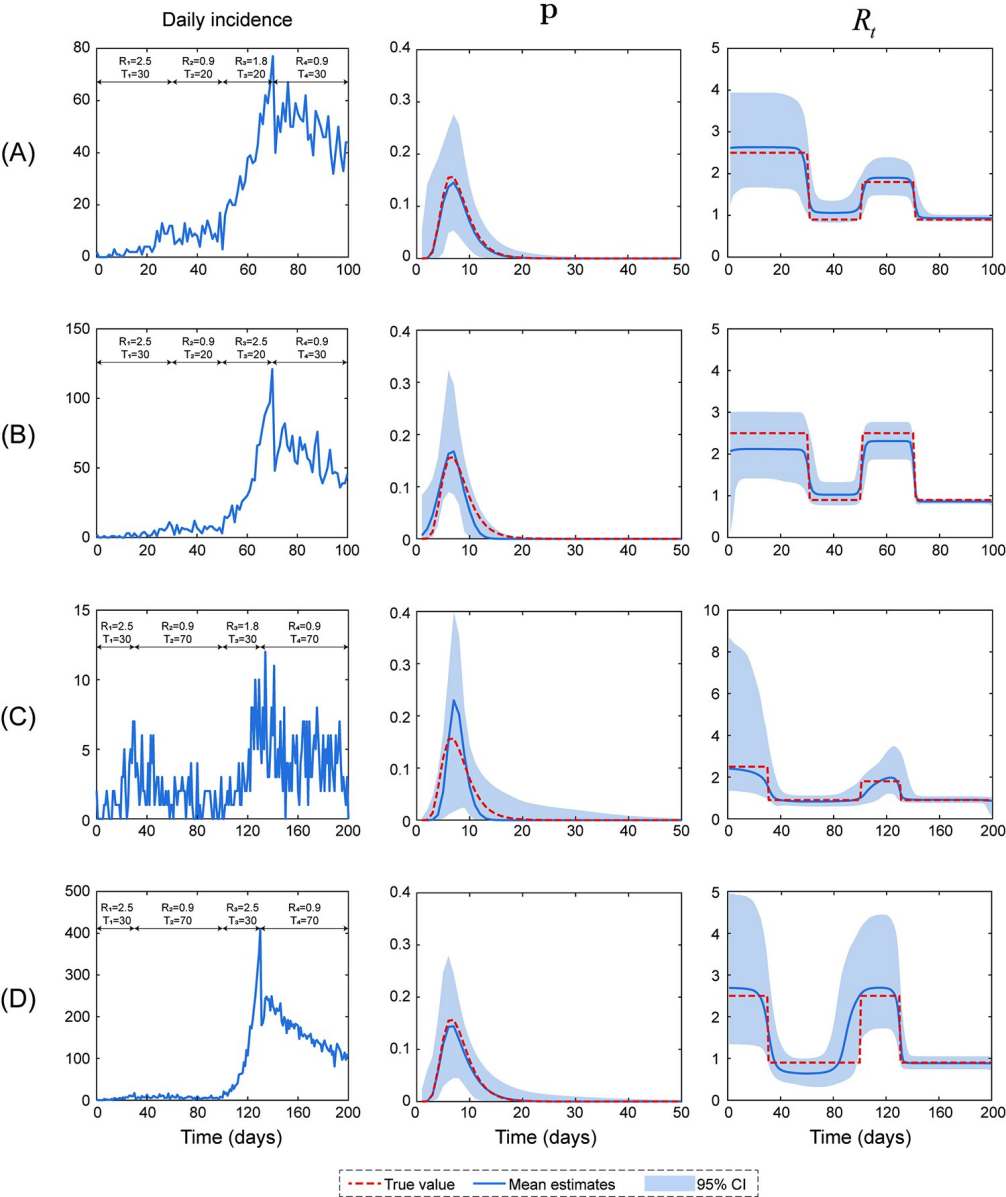
Four examples in which an outbreak was first controlled, then rebounded and was finally controlled again were used for the simulation study in scenario two. Simulations were performed based on the assumptions that the number of initial cases was 2 and the serial interval exhibited a lognormal distribution with a mean and variance of 8 and 9, respectively. Each epidemic was split into four stages, and *R*_1_−*R*_4_ and *T*_1_−*T*_4_ are key parameters that determine the epidemic severity and end time, respectively. The left, middle and right panels present the daily incidence and estimates of the serial interval and instantaneous reproductive number, respectively. The blue lines show the estimates, and the light blue area denotes their 95% credible intervals. The red dashed lines represent the ground truth.

**Fig 6:**
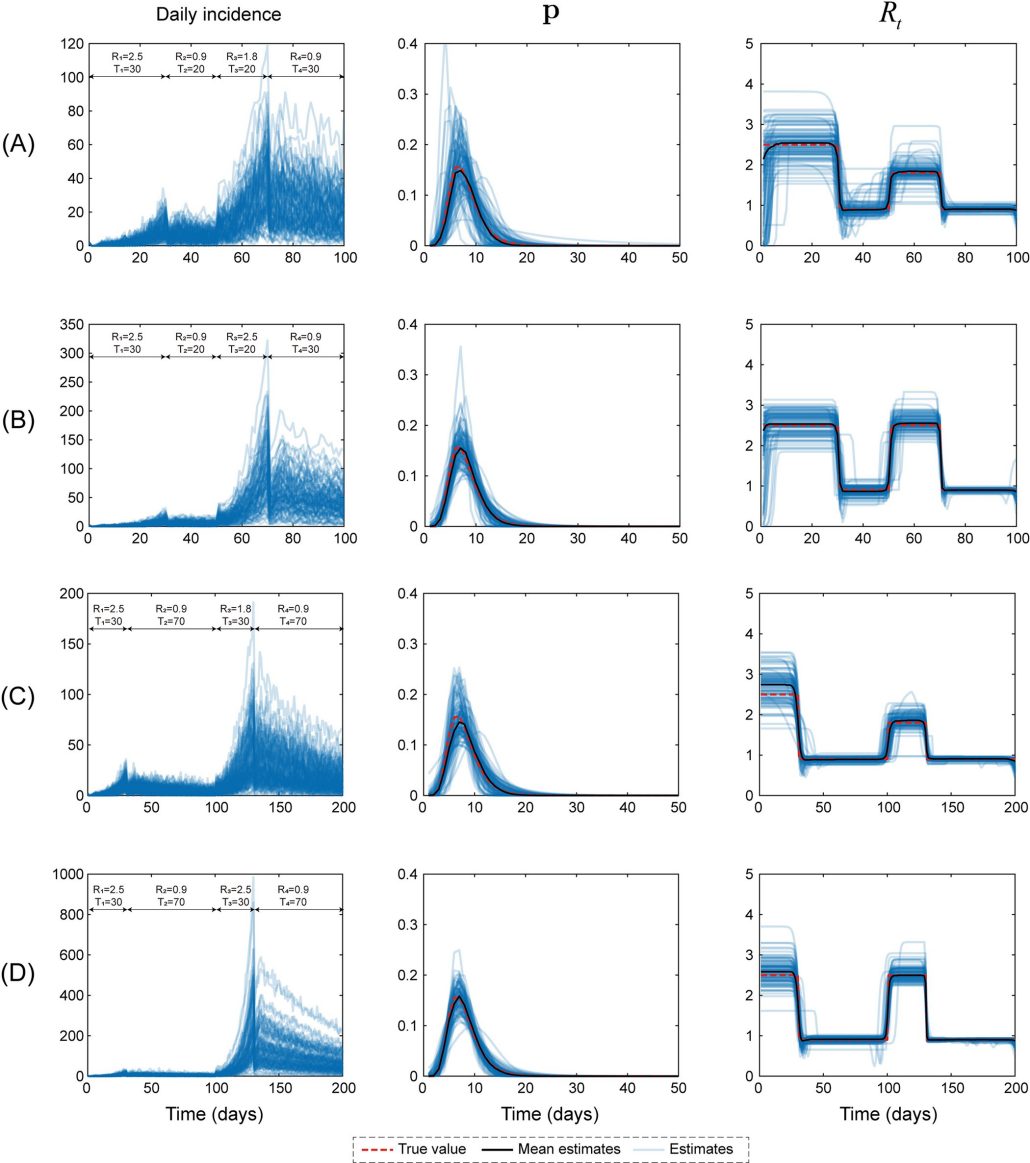
A total of 100 trials were conducted to further verify the performance of our method in scenario two. Simulations were performed based on the assumptions that the number of initial cases was 2 and the serial interval exhibited a lognormal distribution with a mean and variance of 8 and 9, respectively. Each epidemic was split into four stages, and *R*_1_−*R*_4_ and *T*_1_−*T*_4_ are key parameters that determine the epidemic severity and end time, respectively. The left, middle and right panels present the daily incidence and estimates of the serial interval and instantaneous reproductive number, respectively. The blue lines show the estimates, and the dark lines denote their mean values after 100 simulations. The red dashed lines represent the ground truth.

We next analyzed a more complicated epidemic scenario in which an outbreak was first controlled, then rebounded and was finally controlled again. Different choices of parameters, including epidemic severity (*R*_1_, *R*_2_, *R*_3_, and *R*_4_) and duration (*T*_1_, *T*_2_, *T*_3_, and *T*_4_), can lead to distinct epidemic modes (Fig 5, left panels). From each time series of daily incidence, we computed the serial interval and instantaneous reproductive number, and the results were illustrated in the middle and right panels of Fig 5. Our method successfully estimated **p** and *R*_*t*_: all dashed lines of the ground truth were within the 95% CI (Fig 5, light blue area). One hundred trials were randomly selected to further verify the performance of our method in all possible epidemics simulated with the given parameters (*R*_1_−*R*_4_, and *T*_1_−*T*_4_). As shown in Fig 6, the serial interval and instantaneous reproductive number can be calculated faithfully because the mean estimates of both curves agreed well with their own ground truth.

To clarify the impact of time length of the data on the performance of our method in this complicated case, we analyzed the same simulated data sets as Fig 6C by varying the time length from 40 to 200 (S5 Fig). Similar to the results in Fig 3, we found that a longer time length yielded better performance, such as lower *ΔR*_*t*_, *Δμ* and *Δσ*. Besides, we found that if *R*_*t*_ remained unchanged in the next 20 days, our method accurately predicted its trend (the first and fourth rows in S5A Fig); otherwise, our method required additional time to improve the accuracy (the third row in S5A Fig).

### 3.3. Historical epidemic data sets

**Fig 7:**
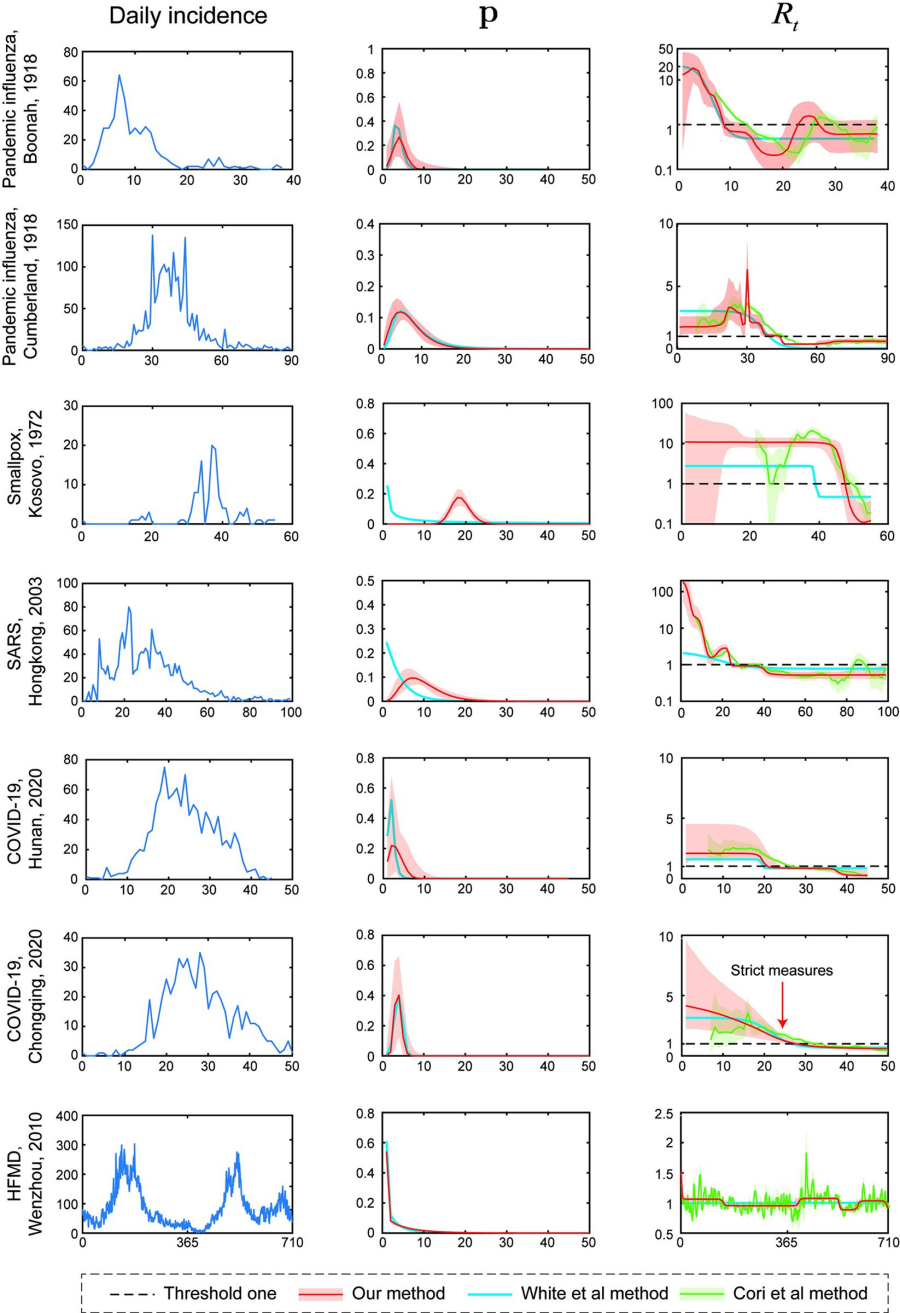
Application of our method to seven historical epidemics. The first column shows daily epidemic curves (from top to bottom) for the pandemic influenza in Boonah, 1918; the pandemic influenza in Cumberland, 1918; the smallpox in Kosovo, February–April 1972; the SARS in Hong Kong, February–June 2003; the COVID-19 in Hunan, 2020; the COVID-19 in Chongqing, 2020; and HFMD in Wenzhou, 2010–2011. The second and third columns show estimates of the serial interval **p** and instantaneous reproductive number *R*_*t*_, respectively. The red lines and light red area, the blue lines, the green lines and the light green area in the middle and right columns denote the estimates obtained using our method, White et al method and Cori et al method, respectively. The dark dashed lines represent the threshold of one, and the red arrow denotes the onset of strict measures.

The estimated serial interval and *R*_*t*_ were obtained for seven historical epidemics from 1918 to 2020 using our proposed method and the methods developed by White et al. and Cori et al., and the results are presented in Fig 7.

#### 3.3.1. Pandemic influenza for the Boonah ship, 1918

We first considered data from a troop ship that embarked in the late fall of 1918. White et al. previously documented the effectiveness of their method in this simple outbreak-control epidemic and showed that the serial interval was 3.81±1.12 [1]. In Fig 7, a large initial *R_t_* (>10) was apparent for the Boonah ship, which was consistent with the results reported by White et al. [1]. A previous study attributed this result to missing data at the beginning of the epidemic; thus, our method reported that a few initially infected individuals resulted in a large number of cases. Several days later, *R_t_* rapidly decreased to sub-epidemic levels, indicating that many susceptible individuals were first infected and then the transmission decreased. We also observed a consistent trend using the three methods, but White et al method failed to detect the small peak around day 28. Furthermore, consistent with the published results [1], the serial interval calculated using our method was 4.00±1.35.

#### 3.3.2. Pandemic influenza in Cumberland, 1918

We now considered another influenza outbreak in 1918. Based on the daily incidence (Fig 7, the panel in the first column, second row), we observed a sharp increase on day 30, with a median estimate of 6.32 (95% CI: 4.80–8.67). *R*_*t*_ then fell below 1 after day 44 and this trend persisted until the end of the outbreak. White et al and Cori et al methods produced the same tendency as our method (Fig 7, the panel in the last column, second row). For the serial interval, our estimate (7.14±3.86) was consistent with White et al (8.28±5.00). Thus, the first two cases of pandemic influenza verified the effectiveness of our method for the simple outbreak-control situation using historical data.

#### 3.3.3. Smallpox in Kosovo, 1972

We then analyzed data from the smallpox outbreak. Here, for a small epidemic with a long mean serial interval, *R*_*t*_ initially had a high value of 10.9 (95% CI: 0–57.6) and this high level was maintained for 40 days. Then, *R*_*t*_ continued to decrease and eventually decreased to the threshold of one on day 48. We observed the same trend for the two other methods, but the turning point (day 40) of White et al method was earlier than that of the two other methods. In addition, our method provided the same results for the serial interval with previously reported values (Table 2), but White et al method underestimated the mean of serial interval (12.06±13.39).

#### 3.3.4. SARS in Hong Kong, 2003

For the SARS outbreak in Hong Kong in 2003, *R*_*t*_ dramatically decreased before day 15 and then slightly rebounded to 2.14 (95% CI: 1.60–2.82) on day 23. Several days later, *R*_*t*_ decreased below the threshold of one, and the epidemic was finally controlled. A similar trend was observed for Cori et al method, while White et al method produced a slightly lower *R*_*t*_ before day 20. For the serial interval, the reported value was consistent with our estimate (8.4±3.8 vs. 9.70±4.57), but White et al. reported a lower value (3.89±3.25 vs. 8.4±3.8).

#### 3.3.5. COVID-19 in Hunan, 2020

We next analyzed a recent pandemic, COVID-19 in Hunan. *R*_*t*_ was clearly divided into two phases. In the first phase (from day 1 to 19), *R*_*t*_ was higher than the threshold of one, indicating an outbreak. In the second phase (from day 20 to 45), *R*_*t*_ decreased below the threshold of one, representing effective control of the epidemic. The results of White et al method was consistent with our estimate, while Cori et al method delayed the second phase. The serial interval estimated by our method and White et al method were 3.42±1.48 and 1.98±0.84, respectively, which were slightly lower than the previously reported value of 4.40±3.17 [26].

#### 3.3.6. COVID-19 in Chongqing, 2020

Another COVID-19 outbreak was then analyzed. On January 1, 2020 (day 0), the first case was imported to Chongqing, and as of February 24, 2020, a total of 576 cases of COVID-19 were confirmed. The initial outbreak led to a high *R*_*t*_ (4.15 with 95% CI 2.25–9.53). On January 24, 2020 (day 24), the local government gradually implemented and strengthened prevention and control measures for the epidemic. As a result, the reproductive number dropped below one on day 28 and continued to decrease until the end of the epidemic. At the same time, the other two methods reported the same trend. In this case, our method (3.79±0.86 vs. 5.2±6.2) and White et al method (3.89±0.97 vs. 5.2±6.2) both obtained lower estimates for the serial interval.

#### 3.3.7. HFMD in Wenzhou, 2010–2011

HFMD was finally analyzed to illustrate the effectiveness of our method for complicated epidemic curves. We found three peaks in daily incidence (Fig 7), and each peak coincided with a period of *R*_*t*_>1. The first period occurred from day 1 to 146, with a median estimate of 1.07; the second outbreak occurred from day 403 to 541, with a median of 1.07; and the last outbreak occurred from day 611 to 701, with a median of 1.03. Cori et al method reported the same trend, while White et al method failed to estimate *R*_*t*_. Our method yielded a similar result for the serial interval (3.86±5.08 vs. 3.7±2.6) as a previous report [27], further illustrating its robust performance, while White et al method reported a lower serial interval (2.39±2.69 vs. 3.7±2.6).

## 4. Discussion

We developed a new framework to simultaneously estimate the instantaneous reproductive number and serial interval using only the time series of daily incidence. Our approach built on a well-established approach [1,7] and addressed a crucial drawback that many studies have faced. The distinct feature of our framework is that the serial interval distribution and instantaneous reproductive number can be estimated jointly from the latest available data without including additional contact tracing data in the estimation procedure. By incorporating AWN interpolation and BIC, our method precisely estimated transmissibility and described the timing of an intervention and the rapidity with which it affects transmission. In addition, we provided four alternative distributions used for serial interval estimation to further maximize the likelihood function (Eq 1). More importantly, the MCMC method provided a reliable result for determining the distribution of the instantaneous reproductive number and serial interval, which further helped us to understand the variable range of an epidemic. The results for two artificial scenarios and seven historical epidemics suggest that our method has promising advantages for application in estimating transmissibility.

White et al employed a likelihood-based methodology tailored to a two-phase epidemic to estimate the serial interval and the instantaneous reproductive number for each day of an epidemic and then applied this method to data from the 1918 influenza outbreak [1]. In artificial scenario one, this method provided reliable results for estimating these quantities (Fig 4). By extending the statistical framework reported previously [7], the current study provides an alternative and improved method for estimating these quantities for multiphase epidemics. First, our method provided comparable results to those obtained using White et al method for a two-phase outbreak. Second, the reliable estimation results of the instantaneous reproductive number for four-phase epidemics (artificial scenario two, Figs 5 and 6) further extended the application of our method. In contrast to White et al method, which is tailored to a two-phase epidemic, our method automatically divided the epidemic into *n* stages via AWN interpolation, and *n* was determined using BIC. Thus, our method can estimate any shape of the curve for instantaneous reproductive number.

The most commonly used approach to estimate instantaneous reproductive number is Cori et al method [6]. The first point that hinders its popularity is that it uses pre-existing estimates of the serial interval distribution as an input. This approach potentially leads to delays between inferring the serial interval and subsequent estimation of transmissibility or indicates that estimates of *R*_*t*_ are based on estimates of the serial interval from earlier outbreaks. However, it may not be accurate when emerging infectious diseases with unknown natural history outbreaks. Secondly, *R*_*t*_ estimated by Cori et al method did not change immediately after interventions were applied (Fig 4C) due to the sliding window used; instead, it changed smoothly in a delayed manner. In contrast, our method reported that *R*_*t*_ changed immediately without delay and produced lower *ΔR*_*t*_ values compare with Cori et al method (Fig 4D). Thus, an estimate of Cori et al method may not be the ideal choice for policymakers who wish to understand the effects of control interventions.

Our method may be promising when serial interval information is missing. First, we observed a continuous curve for the instantaneous reproductive number over time due to AWN interpolation, which provided a clear illustration of current control measures for policymakers (Fig 7). Second, the estimates of the serial interval were much more accurate than those using White et al method (Table 2). Last, the instantaneous reproductive number changed immediately after the intervention. For instance, after the local government implemented strict measures on day 24 (Fig 7, COVID-19 in Chongqing), the instantaneous reproductive number rapidly decreased below the threshold 3 days later.

We next reported the reason why the distribution of serial interval was estimated instead of generation time. Serial interval is the time between onset of symptoms of a case and onset of symptoms of his/her secondary cases, while generation time refers to the time from the infection of a primary case to infection of the cases he/she generates. Thus, generation time differs from serial interval. We applied our method to data consisting of daily counts of symptom onset where the infectivity profile **p** was approximated from the distribution of the serial interval because the timing of symptom onset is usually known, and such data collected in closed settings can reliably be ascertained after an epidemic. However, time of infection are rarely observed, and the generation time distribution is therefore difficult to measure and validate.

In addition to interventions, other factors may affect the instantaneous reproductive number. Firstly, the results obtained using our method may be sensitive to the time length during the epidemic. This result is clearly presented in Fig 3 and S5 Fig, in a longer time length, particularly a value greater than 40, yielded better performance, including lower *ΔR_t_*, *Δμ* and *Δσ* values. Therefore, it should be carefully used especially in the early stage of the epidemic. Secondly, our method may be feasible to predict unchangeable transmissibility. Due to the nature of interpolation, our method performed well when *R*_*t*_ remained unchanged over the next 20 days (Fig 3 and S5). However, when predicting sharp changes in *R*_*t*_, our method requires more information to improve the accuracy. Thirdly, serial interval that changed over time had a trivial impact on the *R*_*t*_ estimation. Serial interval has been shown to change over time, and this change is driven by enhanced nonpharmaceutical interventions, particularly case isolation [25,35]. Our simulation results showed that even the use of a fixed serial interval did not affect the final result of the *R*_*t*_ estimation (S4 Fig), which largely expands the application of our method. The underlying reason of unbiased estimates of *R*_*t*_ (S4C Fig) may attributed to that our method simultaneously estimates the serial interval and instantaneous reproductive number, which provides the possibility that the estimated serial interval fits the true serial interval distribution that was averaged over time well. Lastly, seasonal variations in the parameters governing disease spread play a crucial role in the transmission of many pathogens. For example, the transmission of HFMD varies due to factors including temperature, relative humidity, and school opening [17]. Our method successfully estimated the instantaneous reproductive number and serial interval based on the daily incidence. We observed that the instantaneous reproductive number in the spring semester was higher than the threshold of one, while its value in the fall semester was lower than the threshold, which depicted seasonal variation.

A previous study showed that an increasing epidemic size led to improvements in the reproductive number estimate [7]. The serial interval distribution, *μ*, *σ*^2^, *N*_0_, *R*_1_, and *R*_2_ are key parameters that impact the epidemic size. We thus first tested our method by varying these epidemic parameters. Increasing the number of initial cases, decreasing the mean serial interval, and increasing the epidemic severity (*R*_1_) of a particular disease decreased *ΔR*_*t*_, *Δμ* and *Δσ* (Fig 3B, 3D and 3E) because these parameters may substantially affect the scale of an epidemic. In contrast, the degree of control measures (*R*_2_), variance and mean of the serial interval, and serial interval distribution exerted a noninfluential impact on *ΔR*_*t*_; the underlying reason was that these factors may not be significantly associated with the epidemic size (Fig 3A, 3C and 3F). This evidence may partially explain the varying performance of our method under different conditions.

However, the present study has some limitations that should be addressed in future studies. Firstly, we assumed that secondary cases were generated in a Poisson distribution according to previous studies [1,6,7,13]. This assumption may not be accurate because it was not verified by real data. In fact, infectious diseases may have different characteristics that would lead primary infectors to generate secondary cases at varying rates, which may produce many possible distributions and increase the difficulty of estimation outcomes. Although this approach may not be perfectly accurate for disease generation, this assumption simplified the estimation process. Secondly, the usefulness was not assessed by determining the effect of right truncation. Due to different delays, the date of infection cases cannot be reported in a timely manner, and thus, reported cases in real or near-real time are systematically right-censored [36]. Thirdly, this study did not address the issue of the imperfect observation of cases. Numerous studies have shown that reported cases particularly in the beginning of an epidemic, are commonly biased [36–38], and only the most severe cases are reported, or diagnostics are weighted differently across subpopulations or age groups. Thus, our method should be carefully applied if the distribution of ascertainment and reporting rates are not distributed homogeneously over time, which may lead to severely biased estimates. Fourthly, the interchangeability between the serial interval and the generation time was not assessed, and *R*_*t*_ calculated from serial interval may suffer from bias. A previous study has showed the relationship between serial interval and generation time, and the latter can be estimated from the former but with inaccurate variance [39]. Further study should be implemented to directly estimate generation time with highly accurate variance, and improve the accuracy of *R*_*t*_ estimation. Fifthly, we also assumed that serial interval was assumed to be positive [6], but it can sometimes take negative values, especially for COVID-19 virus infection. Lastly, from a public health/surveillance perspective, interventions, such as case isolation, can shorten the mean serial interval and change the instantaneous reproductive number, and then may pose challenges to our method. Despite the high performance our method achieved when serial interval changed over time (S4 Fig), serial interval can vary over time during the epidemic due to sampling bias and additional studies should be conducted in the future to construct a model of this issue.

## 5. Conclusions

In this study, we developed a new framework to jointly estimate the instantaneous reproductive number and serial interval by relying only on the time series of daily incidence. The simulation study revealed that our estimates of the two quantities were consistent with the ground truth. Seven data sets of historical epidemics further verified the robust performance of our method, and our analysis was also in agreement with previously published results. Without the need for additional contact tracing data, our simple method will be helpful for the timely analysis of new outbreaks and the retrospective study of historical epidemics. Further studies are required to verify the robustness of our method, especially surveillance data.

## Supporting information

S1 Text Additional information on model specification. The file contains four sections: (1) White et al method, (2) simplification of the likelihood, (3) model for generating simulation data, (4) the gradient of the negative log-likelihood −ln *L*(*R*_*t*_, **p**|***N***).
(DOCX)

S2 Text Comparative methods. The file includes White et al method and Cori et al method.
(DOCX)

S1 Table Prior information of parameters for MCMC method based on MATLAB toolbox. (DOCX)

S2 Table Comparison of standard deviation of serial interval between White et al and our method. (DOCX)

S1 Fig MCMC results of each parameter. MCMC chains (A), distributions (B), and correlation matrix (C) of all nine parameters. Results of Geweke convergence diagnostic method were shown in the top of each chain (A), and P>0.05 was diagnosed as convergent chain. The MCMC algorithm ran for 2×10^6^ iterations with a burn-in of 10^6^ iterations. MCMC: Markov Chain Monte Carlo.
(DOCX)

S2 Fig Comparison between the results of BIC and AIC. *ΔR*_*t*_. (A), *Δμ* (B), *Δσ* (C), and the number of key variables {*t*_*j*_, *r*_*j*_}, *j* = 1,2,⋯,*n* used for *R*_*t*_ interpolation (D). Compared to the AIC, BIC required significantly a lower number of key variables for *R*_*t*_ interpolation, meaning that BIC tended to select less complex model. Meanwhile, BIC was a more effective penalized model selection criteria since it produced remarkably lower *ΔR*_*t*_, *Δμ*, and *Δσ*, when compared with AIC. A total of 100 trials were conducted to investigate AIC and BIC results. Simulations were performed based on the assumptions that the number of initial cases was 2, the serial interval exhibited a lognormal distribution with a mean and variance of 8 and 9, respectively, and a constant R before (*R*_1_ = 2.5) and after (*R*_2_ = 0.9) a control measure on day 40. AIC: Akaike information criterion; BIC: Bayesian information criterion. *: P<0.05, and ***: P<0.001.
(DOCX)

S3 Fig The performance of our method when varying different parameters. (A) Serial interval distribution. (B) Mean value of the serial interval. (C) Standard deviation of the serial interval. (D) The number of initial cases. (E) The epidemic severity *R*_1_. (F) The effectiveness of control measures *R*_2_. Simulations were performed based on the assumptions that the number of initial cases was 2, the serial interval exhibited a lognormal distribution with a mean and variance of 8 and 9, respectively, and a constant R before (*R*_1_ = 2.5) and after (*R*_2_ = 0.9) a control measure on day 40. When we varied a parameter to study its effects, the others were kept unchanged. *: P<0.05, **: P<0.01, and ***: P<0.001.
(DOCX)

S4 Fig The effects of changing serial interval on the results. (A) Distribution of serial interval over time. (B) Daily incidence generated by varying the serial interval. (C) Estimates of instantaneous reproductive number. (D) Estimates of serial interval. The red dashed curve denotes the true serial interval distribution averaged over time. (E) *ΔR*_*t*_. (F) *Δμ* is the absolute error between the true mean serial interval averaged over time and our estimates. (G) *Δσ* is the absolute error between the true standard deviation of the serial interval averaged over time and our estimates. A total of 100 trials were conducted to investigate the performance of our method by varying mean serial interval (from 8 to 3) and fixed mean serial interval (8). Simulations were performed based on the assumptions that the number of initial cases was 2, the serial interval exhibited a lognormal distribution with a variance of 9, and a constant R before (*R*_1_ = 2.5) and after (*R*_2_ = 0.9) a control measure on day 40. *: P<0.05, and ***: P<0.001.
(DOCX)

S5 Fig The effects of time length on the results. A total of 100 trials were conducted to investigate the performance of our method by varying the time length (from 40 to 200). Simulations were performed based on the assumptions that the number of initial cases was 2, the serial interval exhibited a lognormal distribution with a mean and variance of 8 and 9, respectively, and it was split into four stages. The left, middle and right panels are the estimates of instantaneous reproductive number, the estimates of the serial interval and the results of the three indices, respectively. The blue lines show the estimates, and the dark lines denote their mean values after 100 simulations. Additionally, the gray lines denote predictions and dashed dark lines represent their mean values after 100 simulations. The red dashed lines represent the ground truth. *: P<0.05, **: P<0.01, and ***: P<0.001.
(DOCX)

S1 Codes MATLAB main model file. This MATLAB script (main_an_example.m) provided an example illustrating the workflow of our method.
(ZIP)
