# Supplementary material for: A new method for the joint estimation of instantaneous reproductive number and serial interval during epidemics
Source: PLoS Comput Biol. 2023 Mar 31;19(3):e1011021. doi: 10.1371/journal.pcbi.1011021 (PMC10096265; doi:10.1371/journal.pcbi.1011021)
Supplement: S1 Text — The file contains four sections: (1) White et al method, (2) simplification of the likelihood, (3) model for generating simulation data, (4) the gradient of the negative log-likelihood −ln L(Rt, p|N). (DOCX) [file pcbi.1011021.s001.docx]

**S1 Text**

**Additional information on model specification**

**Section S1. White et al method**

White et al. presented a method for the simultaneous estimation of the basic reproductive number, $R_{0}$, and the serial interval $\mathbf{p}$ for infectious disease epidemics, using the daily number of new cases $\mathbf{N}=\{N_{t}\},t=0,1,2\cdots T$ [1]. It has a likelihood function

$L(R_{0},\mathbf{p}|\mathbf{N})=\prod_{t=1}^{T} \frac{e^{-\mu_{t}}(\mu_{t})^{N_{t}}}{N_{t}!},$ (S1)

where $\mu_{t}=R_{0}(\sum_{i=1}^{k} N_{t-i}p_{i})$. From this likelihood, the authors derived maximum likelihood estimates, and simultaneously estimated $R_{0}$ and $\mathbf{p}$.

**Section S2. Simplification of the likelihood**

White et al [1] presented a method for the simultaneous estimation of the basic reproductive number, $R_{0}$, and the serial interval for infectious disease epidemics, using the number of new cases per day. $X_{t,.}$ was used in their study and was assumed to follow a multinomial distribution. The likelihood function was summarized as follows:

$L(R_{0},\mathbf{p}|\mathbf{N},\mathbf{X})=\prod_{\tau=1}^{t} \left[\frac{e^{-N_{\tau}R_{0}}(N_{\tau}R_{0})^{X_{\tau,.}}}{X_{\tau,.}!}\right]\left[\left(\begin{matrix} X_{\tau,.} \\ X_{\tau,\tau+1}\cdots X_{\tau,\tau+k} \end{matrix}\right)p_{1}^{X_{\tau,\tau+1}}\cdots p_{k}^{X_{\tau,\tau+k}}\right].$ (S2)

The authors rearranged the terms in this likelihood function such that the future $X_{t,t+i}(i>0)$ was properly normalized and summed as Poisson random variables. Arranging the rest of the terms allowed them to sum the remaining unobserved $X_{t,t+i}$ as binomial and multinomial random variables. The likelihood was then reduced to a thinned Poisson distribution:

$L(R_{0},\mathbf{p}|\mathbf{N})=\prod_{\tau=1}^{t} \frac{e^{-\mu_{\tau}}\mu_{\tau}^{N_{\tau}}}{N_{\tau}!},$ (S3)

where $\mu_{t}=R_{0}(\sum_{i=1}^{k} N_{t-i}p_{i})$.

Based on the aforementioned likelihood, Cori et al. [2] presented a method to estimate $R_{t}$ when the distribution of serial interval and daily reported cases is available. The likelihood function that Cori et al. used is similar to that used by White et al [1], but incorporated different reproductive numbers:

$L(R_{t},\mathbf{p}|\mathbf{N})=\prod_{\tau=1}^{t} \frac{e^{-R_{\tau}\mu_{\tau}}(R_{\tau}\mu_{\tau})^{N_{\tau}}}{N_{\tau}!},$ (S4)

where $\mu_{t}=\sum_{i=1}^{\min(k,t)} N_{t-i}p_{i}$. For simplicity, we adopted the same likelihood function as that using by Cori et al. to avoid collecting unobserved data $X_{t,t+i}$.

**Section S3. Model for generating simulation data**

The model for the simulation is same as the process used to generate the likelihood function and can be briefly summarized in the following four steps:

**Step one**: We need the number of initial cases $N_{0}$, the instantaneous reproductive number $R_{t}$, the serial interval distribution $\mathbf{p}$, the maximal length of the serial interval *k*, and the maximum observation day *T*.

**Step two**: On day 0, $N_{0}$ cases generated a random number $X_{0,.}=\sum_{i=1}^{k} X_{0,0+i}$ of Poisson distribution $P(N_{0}R_{0})$, which is the total number of cases from day 1 to day *i*. Then, $\mathbf{X}_{\mathbf{0}}=\{X_{0,0+1},X_{0,0+2},\cdots,X_{0,0+k}\}$ were randomly produced by a multinomial distribution with parameters $(X_{0,.},\mathbf{p},k)$. Thus, we obtained the total number of cases $N_{1}$ on day 1, and the number of cases $X_{0,0+i}$ that appeared on day *i* that are infected by individuals who develop symptoms on day 0.

**Step three**: On day *t*, the number of cases $N_{t}$ was calculated using the formular $N_{t}=\sum_{i=t-\min(t,k)}^{t-1} X_{it}$. Similar to Step two, $N_{t}$ generated the total number of cases $X_{t,.}=\sum_{i=1}^{k} X_{t,t+i}$ and the number of cases $X_{t,t+i}$ on day *t*+*i*.

**Step four**: Step three was repeated until *t*=*T*. Then, we obtained the number of new cases $\mathbf{N}=\{N_{t}\},t=0,1,2\cdots T$ from day 0 to *T* of the epidemic for the simulation.

**Section S4. The gradient of the negative log-likelihood** $-\ln L(R_{t},\mathbf{p}|\mathbf{N})$

The gradient of $-\ln L(R_{t},\mathbf{p}|\mathbf{N})$ to $a$,

$\frac{\partial\left(-\ln L(R_{t},\mathbf{p}|\mathbf{N})\right)}{\partial a}=-\sum_{t=1}^{T} \left(\frac{N_{t}}{\Omega_{t}}-R_{t}\right)\frac{\partial\Omega_{t}}{\partial a},$ (S5)

$\frac{\partial\Omega_{t}}{\partial a}=\sum_{i=1}^{\min(k,t)} N_{t-i}\frac{\partial p_{i}}{\partial a},$ (S6)

$\frac{\partial p_{i}}{\partial a}=\frac{1}{\sum_{j=1}^{k} \psi_{j}}\frac{\partial\psi_{i}}{\partial a}-\frac{\psi_{i}}{\left(\sum_{j=1}^{k} \psi_{j}\right)^{2}}\sum_{j=1}^{k} \frac{\partial\psi_{j}}{\partial a},i=1,2,\cdots,k,$ (S7)

$\frac{\partial\psi_{i}}{\partial a}=\int_{i-1}^{i} \frac{\partial f(x|a,b)}{\partial a}dx,i=1,2,\cdots,k.$ (S8)

The gradient of $-\ln L(R_{t},\mathbf{p}|\mathbf{N})$ to $b$,

$\frac{\partial\left(-\ln L(R_{t},\mathbf{p}|\mathbf{N})\right)}{\partial b}=-\sum_{t=1}^{T} \left(\frac{N_{t}}{\Omega_{t}}-R_{t}\right)\frac{\partial\Omega_{t}}{\partial b},$ (S9)

$\frac{\partial\Omega_{t}}{\partial b}=\sum_{i=1}^{\min(k,t)} N_{t-i}\frac{\partial p_{i}}{\partial b},$ (S10)

$\frac{\partial p_{i}}{\partial b}=\frac{1}{\sum_{j=1}^{k} \psi_{j}}\frac{\partial\psi_{i}}{\partial b}-\frac{\psi_{i}}{\left(\sum_{j=1}^{k} \psi_{j}\right)^{2}}\sum_{j=1}^{k} \frac{\partial\psi_{j}}{\partial b},i=1,2,\cdots,k,$ (S11)

$\frac{\partial\psi_{i}}{\partial b}=\int_{i-1}^{i} \frac{\partial f(x|a,b)}{\partial b}dx,i=1,2,\cdots,k.$ (S12)

The gradient of $-\ln L(R_{t},\mathbf{p}|\mathbf{N})$ to $c$,

$\frac{\partial\left(-\ln L(R_{t},\mathbf{p}|\mathbf{N})\right)}{\partial c}=-\sum_{t=1}^{T} \left(\frac{N_{t}}{R_{t}}-\Omega_{t}\right)\frac{\partial R_{t}}{\partial c},$ (S13)

$\frac{\partial R_{t}}{\partial c}=\sum_{i=1}^{n} r_{i}\frac{\partial w_{i}}{\partial c},$ (S14)

$\frac{\partial w_{i}}{\partial c}=\left(\sum_{j=1}^{n} d_{j}\right)^{-1}\frac{\partial d_{i}}{\partial c}-\left(\sum_{j=1}^{n} d_{j}\right)^{-2}d_{i}\left(\sum_{j=1}^{n} \frac{\partial d_{j}}{\partial c}\right),$ (S15)

$\frac{\partial d_{i}}{\partial c}=-d_{i}\cdot\left|t-t_{i}\right|\cdot\mathrm{sgn}(c).$ (S16)

The gradient of $-\ln L(R_{t},\mathbf{p}|\mathbf{N})$ to $t_{i}$,

$\frac{\partial\left(-\ln L(R_{t},\mathbf{p}|\mathbf{N})\right)}{\partial t_{i}}=-\sum_{t=1}^{T} \left(\frac{N_{t}}{R_{t}}-\Omega_{t}\right)\frac{\partial R_{t}}{\partial t_{i}},$ (S17)

$\frac{\partial R_{t}}{\partial t_{i}}=\sum_{j=1}^{n} r_{i}\frac{\partial w_{j}}{\partial t_{i}},$ (S18)

$\frac{\partial w_{j}}{\partial t_{i}}=\left(\sum_{l=1}^{n} d_{l}\right)^{-1}\frac{\partial d_{j}}{\partial t_{i}}-\left(\sum_{l=1}^{n} d_{l}\right)^{-2}d_{j}\left(\sum_{l=1}^{n} \frac{\partial d_{l}}{\partial t_{i}}\right),$ (S19)

$\frac{\partial d_{j}}{\partial t_{i}}=\left\{\begin{matrix} d_{j}\cdot\left|c\right|\cdot\mathrm{sgn}(t-t_{j}), & i=j \\ 0, & i\neq j \end{matrix}\right..$ (S20)

The gradient of $-\ln L(R_{t},\mathbf{p}|\mathbf{N})$ to $r_{i}$,

$\frac{\partial\left(-\ln L(R_{t},\mathbf{p}|\mathbf{N})\right)}{\partial r_{i}}=-\sum_{t=1}^{T} \left(\frac{N_{t}}{R_{t}}-\Omega_{t}\right)\frac{\partial R_{t}}{\partial r_{i}},$ (S21)

$\frac{\partial R_{t}}{\partial r_{i}}=w_{i}.$ (S22)

**References:**

1. White LF, Pagano M. A likelihood-based method for real-time estimation of the serial interval and reproductive number of an epidemic. Stat Med. 2008;27: 2999–3016. doi:10.1002/sim.3136

2. Cori A, Ferguson NM, Fraser C, Cauchemez S. A new framework and software to estimate time-varying reproduction numbers during epidemics. Am J Epidemiol. 2013;178: 1505–1512. doi:10.1093/aje/kwt133
