# Supplementary material for: A new method for the joint estimation of instantaneous reproductive number and serial interval during epidemics
Source: PLoS Comput Biol. 2023 Mar 31;19(3):e1011021. doi: 10.1371/journal.pcbi.1011021 (PMC10096265; doi:10.1371/journal.pcbi.1011021)
Supplement: S2 Text — The file includes White et al method and Cori et al method. (DOCX) [file pcbi.1011021.s002.docx]

**S2 Text**

**Comparative methods**

**White et al method**

This method implements a logistic curve that is suitable only for two-stage epidemics, i.e., outbreak and control stages [1]. It has a likelihood function

$L(R_{t},\mathbf{p}|\mathbf{N})=\prod_{t=1}^{T} \frac{e^{-\mu_{t}}(\mu_{t})^{N_{t}}}{N_{t}!},$ (S1)

where $\mu_{t}=R_{t}(\sum_{i=1}^{k} N_{t-i}p_{i})$, $R_{t}$ and $\mathbf{p}=\{p_{1},p_{2},\cdots,p_{k}\}$ are the instantaneous reproductive number and serial interval, respectively. Maximization of this likelihood with respect to $R_{t}$ and $\mathbf{p}$ yields estimates of these parameters. $\mathbf{p}$ was parameterized by allowing it to follow a traditional parametric form for a serial interval (for instance, a Weibull, gamma, lognormal, or exponential distribution). Then, $p_{i}$ is a function of the parameters of the density (for instance, in the case of the gamma distribution, $p_{i}$ depends on only the shape and rate parameters of the gamma distribution).

Similarly, $R_{t}$ was modeled as a function of the four-parameter logistic curve as follows:

$R_{t}=a+\frac{b}{1+e^{c(t-d)}}.$ (S2)

The parameters, *a*, *c* and *d*, represent the final height of the curve, the curvature over the inflection, and the point of inflection, respectively, and *a*+*b* describes the initial height of the curve. These key parameters determine the biological meaning of an outbreak. The initial height represents $R_{t}$ prior to the intervention and rapid depletion of susceptible individuals, and the inflection point and its steepness show the timing of the intervention and its effectiveness. The final height denotes the ultimate value of $R_{t}$, which is typically less than one, indicating a sub-epidemic state of disease transmission.

After maximization of the likelihood (S1) using the Nelder-Mead method with 100 starting values, $\mathbf{p}$ and $R_{t}$ were estimated.

**Cori et al method**

Cori et al. [2] developed a method and software (the EpiEstim R package) for to estimate the instantaneous reproductive number using branching processes and it relies on two inputs: a disease incidence time series (the number of new cases at successive times) and the distribution of serial intervals. They modeled transmission with a Poisson process, so that the rate at which someone infected in time step *t-i* generates new infections in time step *t*, is equal to $R_{t}p_{i}$, where $R_{t}$ is the instantaneous reproductive number at time *t* and $p_{i}$ is a probability distribution (hence summing to 1) describing the average infectiousness profile after infection. Therefore, the incidence at time *t* exhibits a Poisson distribution with a mean $R_{t}\sum_{i=1}^{t} N_{t-i}p_{i}$, and the likelihood of the incidence $N_{t}$ for the reproductive number $R_{t}$ that is conditional on the previous incidences $N_{0},\cdots,N_{t-1}$, is :

$P(N_{t}|N_{0},\cdots,N_{t-1},\mathbf{p},R_{t})=\frac{(R_{t}\mu_{t})^{N_{t}}e^{-R_{t}\mu_{t}}}{N_{t}!}$ (S3)

with $\mu_{t}=\sum_{i=1}^{k} N_{t-i}p_{i}$.

Then, the authors assumed constant $R_{t}$ over a time period $[t-\tau+1,t]$, measured using the reproductive number $R_{t-\tau}$, and the likelihood of the incidence during this time period, $N_{t-\tau+1},\cdots,N_{t}$, for the reproductive number $R_{t-\tau}$ that is conditional on the previous incidences $N_{0},\cdots,N_{t-\tau}$, is:

$P(N_{t-\tau+1},\cdots,N_{t}|N_{0},\cdots,N_{t-\tau},\mathbf{p},R_{t})=\sum_{s=t-\tau+1}^{t} \frac{(R_{t,\tau}\mu_{s})N_{s}e^{-R_{t,\tau}\mu_{s}}}{N_{s}!}.$ (S4)

Using a Bayesian framework with a gamma distribution prior with parameters (*a*,*b*) for $R_{t,\tau}$, they obtained the posterior joint distribution of $R_{t,\tau}$ which follows a gamma distribution with a mean of $\frac{a+\sum_{s=t-\tau+1}^{t} N_{s}}{\frac{1}{b}+\sum_{s=t-\tau+1}^{t} \mu_{s}}$. The authors recommended a gamma prior distribution with a mean of 5 and a standard deviation of 5 (therefore *a*=1, *b*=5).

**References:**

1. White LF, Pagano M. A likelihood-based method for real-time estimation of the serial interval and reproductive number of an epidemic. Stat Med. 2008;27: 2999–3016. doi:10.1002/sim.3136

2. Cori A, Ferguson NM, Fraser C, Cauchemez S. A new framework and software to estimate time-varying reproduction numbers during epidemics. Am J Epidemiol. 2013;178: 1505–1512. doi:10.1093/aje/kwt133
