# Supplementary material for: A new method for the joint estimation of instantaneous reproductive number and serial interval during epidemics
Source: PLoS Comput Biol. 2023 Mar 31;19(3):e1011021. doi: 10.1371/journal.pcbi.1011021 (PMC10096265; doi:10.1371/journal.pcbi.1011021)
Supplement: S1 Table — (DOCX) [file pcbi.1011021.s003.docx]

Table S1. Prior information of parameters for MCMC method based on MATLAB toolbox

| Parameter | Initial value | Minimum | Maximum | Prior mean | Prior standard deviation |
| --- | --- | --- | --- | --- | --- |
| *a* | From Step 4 | 0 | +∞ | 0 | +∞ |
| *b* |  | 0 | +∞ | 0 | +∞ |
| *c* |  | 0 | +∞ | 0 | +∞ |
| $t_{j}$ |  | 0 | *t* | 0 | +∞ |
| $r_{j}$ |  | 0 | +∞ | 0 | +∞ |
