# Supplementary material for: A new method for the joint estimation of instantaneous reproductive number and serial interval during epidemics
Source: PLoS Comput Biol. 2023 Mar 31;19(3):e1011021. doi: 10.1371/journal.pcbi.1011021 (PMC10096265; doi:10.1371/journal.pcbi.1011021)
Supplement: S2 Table — (DOCX) [file pcbi.1011021.s004.docx]

Table S2. Comparison of standard deviation of serial interval between White et al and our method

| Disease | Location | Year of Epidemic | Standard Deviation of Serial Interval, days | |
| --- | --- | --- | --- | --- |
|  |  |  | Our method | White et al method |
| Pandemic influenza | Boonah | 1918 | 1.35(95% CI, 0.82-2.40) | 1.12 |
| Pandemic influenza | Cumberland | 1918 | 3.86(95% CI, 2.83-4.98)* | 5.00 |
| Smallpox | Kosovo | 1972 | 12.32(95% CI, 1.69-3.35)* | 13.39 |
| SARS | Hong Kong | 2003 | 4.57(95% CI, 3.11-6.47) | 3.25 |
| COVID-19 | Hunan | 2020 | 1.48(95% CI, 0.51-3.13) | 0.84 |
| COVID-19 | Chongqing | 2020 | 0.86(95% CI, 0.51-1.69) | 0.97 |
| Hand-foot-mouth disease | Wenzhou | 2010-2011 | 5.08(95% CI, 3.77-6.53)* | 2.69 |

*: P<0.05 compared with White et al method.
