# Supplementary material for: A new method for the joint estimation of instantaneous reproductive number and serial interval during epidemics
Source: PLoS Comput Biol. 2023 Mar 31;19(3):e1011021. doi: 10.1371/journal.pcbi.1011021 (PMC10096265; doi:10.1371/journal.pcbi.1011021)
Supplement: S1 Fig — MCMC chains (A), distributions (B), and correlation matrix (C) of all nine parameters. Results of Geweke convergence diagnostic method were shown in the top of each chain (A), and P>0.05 was diagnosed as convergent chain. The MCMC algorithm ran for 2×10^6^ iterations with a burn-in of 106 iterations. MCMC: Markov Chain Monte Carlo. (DOCX) [file pcbi.1011021.s005.docx]

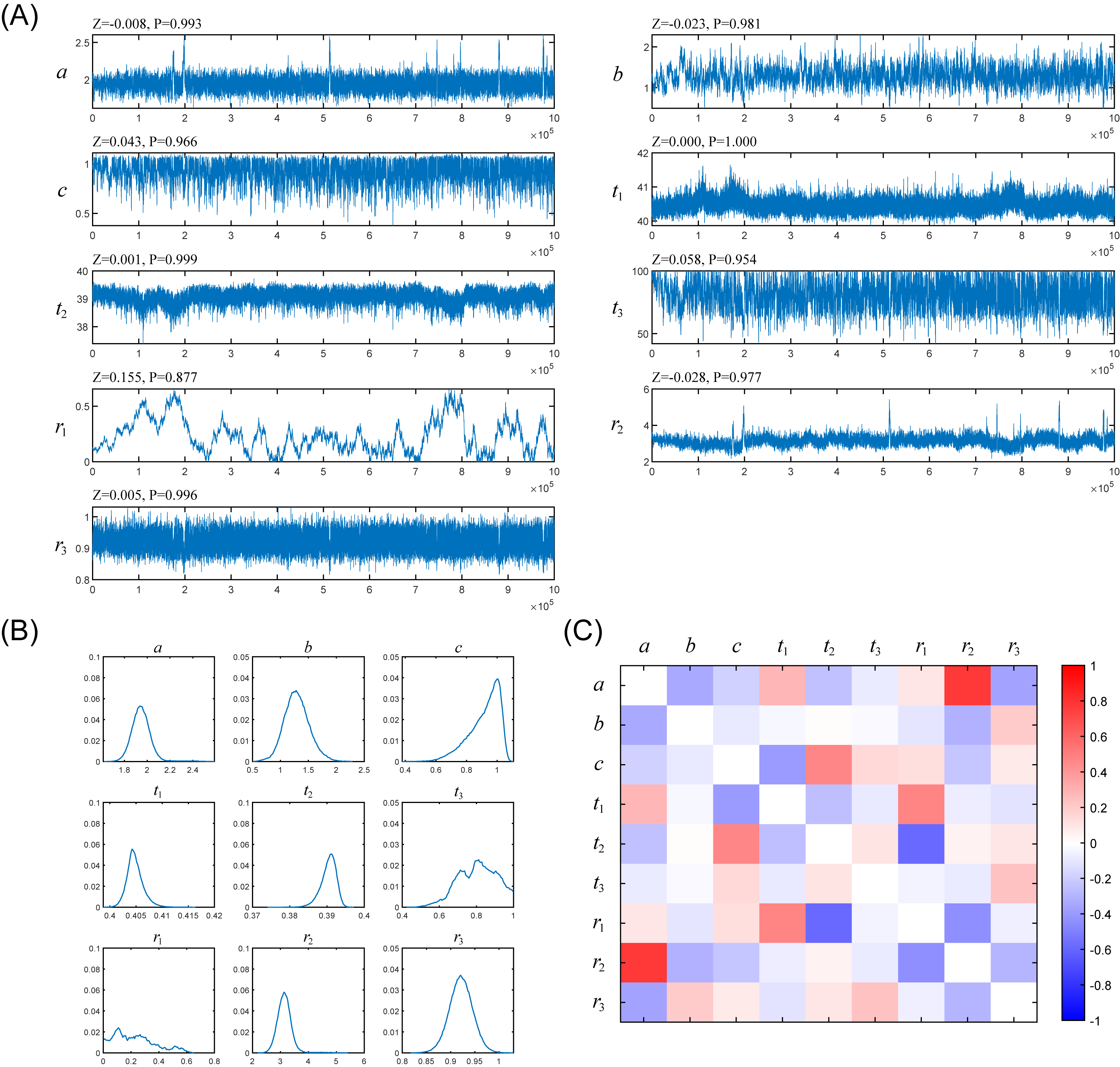


**S1 Fig**. MCMC results for each parameter. MCMC chains (A), distributions (B), and correlation matrix (C) of all nine parameters. Results of Geweke convergence diagnostic method were shown in the top of each chain (A), and P>0.05 was diagnosed as convergent chain. The MCMC algorithm ran for 2×10^6^ iterations with a burn-in of 10^6^ iterations. MCMC: Markov Chain Monte Carlo.
