# Supplementary material for: A new method for the joint estimation of instantaneous reproductive number and serial interval during epidemics
Source: PLoS Comput Biol. 2023 Mar 31;19(3):e1011021. doi: 10.1371/journal.pcbi.1011021 (PMC10096265; doi:10.1371/journal.pcbi.1011021)
Supplement: S2 Fig — (A), Δμ (B), Δσ (C), and the number of key variables {tj, rj}, j = 1,2,⋯,n used for Rt interpolation (D). Compared to the AIC, BIC required significantly a lower number of key variables for Rt interpolation, meaning that BIC tended to select less complex model. Meanwhile, BIC was a more effective penalized model selection criteria since it produced remarkably lower ΔRt, Δμ, and Δσ, when compared with AIC. A total of 100 trials were conducted to investigate AIC and BIC results. Simulations were performed based on the assumptions that the number of initial cases was 2, the serial interval exhibited a lognormal distribution with a mean and variance of 8 and 9, respectively, and a constant R before (R1 = 2.5) and after (R2 = 0.9) a control measure on day 40. AIC: Akaike information criterion; BIC: Bayesian information criterion. *: P<0.05, and ***: P<0.001. (DOCX) [file pcbi.1011021.s006.docx]

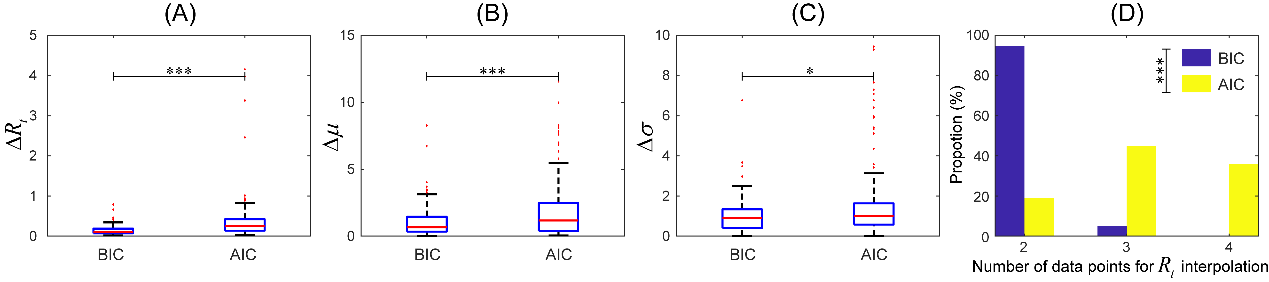


**S2 Fig**. Comparison between BIC and AIC results. $\Delta R_{t}$ (A), $\Delta\mu$ (B), $\Delta\sigma$ (C), and the number of key variables $\{t_{j},r_{j}\},j=1,2,\cdots,n$ used for $R_{t}$ interpolation (D). Compared to the AIC, BIC required significantly a lower number of key variables for $R_{t}$ interpolation, meaning that BIC tended to select less complex model. Meanwhile, BIC was a more effective penalized model selection criteria since it produced remarkably lower $\Delta R_{t}$, $\Delta\mu$, and $\Delta\sigma$, when compared with AIC. A total of 100 trials were conducted to investigate AIC and BIC results. Simulations were performed based on the assumptions that the number of initial cases was 2, the serial interval exhibited a lognormal distribution with a mean and variance of 8 and 9, respectively, and a constant R before (R_1_=2.5) and after (R_2_=0.9) a control measure on day 40. AIC: Akaike information criterion; BIC: Bayesian information criterion. *: P<0.05, and ***: P<0.001.
