# Supplementary material for: A new method for the joint estimation of instantaneous reproductive number and serial interval during epidemics
Source: PLoS Comput Biol. 2023 Mar 31;19(3):e1011021. doi: 10.1371/journal.pcbi.1011021 (PMC10096265; doi:10.1371/journal.pcbi.1011021)
Supplement: S3 Fig — (A) Serial interval distribution. (B) Mean value of the serial interval. (C) Standard deviation of the serial interval. (D) The number of initial cases. (E) The epidemic severity R1. (F) The effectiveness of control measures R2. Simulations were performed based on the assumptions that the number of initial cases was 2, the serial interval exhibited a lognormal distribution with a mean and variance of 8 and 9, respectively, and a constant R before (R1 = 2.5) and after (R2 = 0.9) a control measure on day 40. When we varied a parameter to study its effects, the others were kept unchanged. *: P<0.05, **: P<0.01, and ***: P<0.001. (DOCX) [file pcbi.1011021.s007.docx]

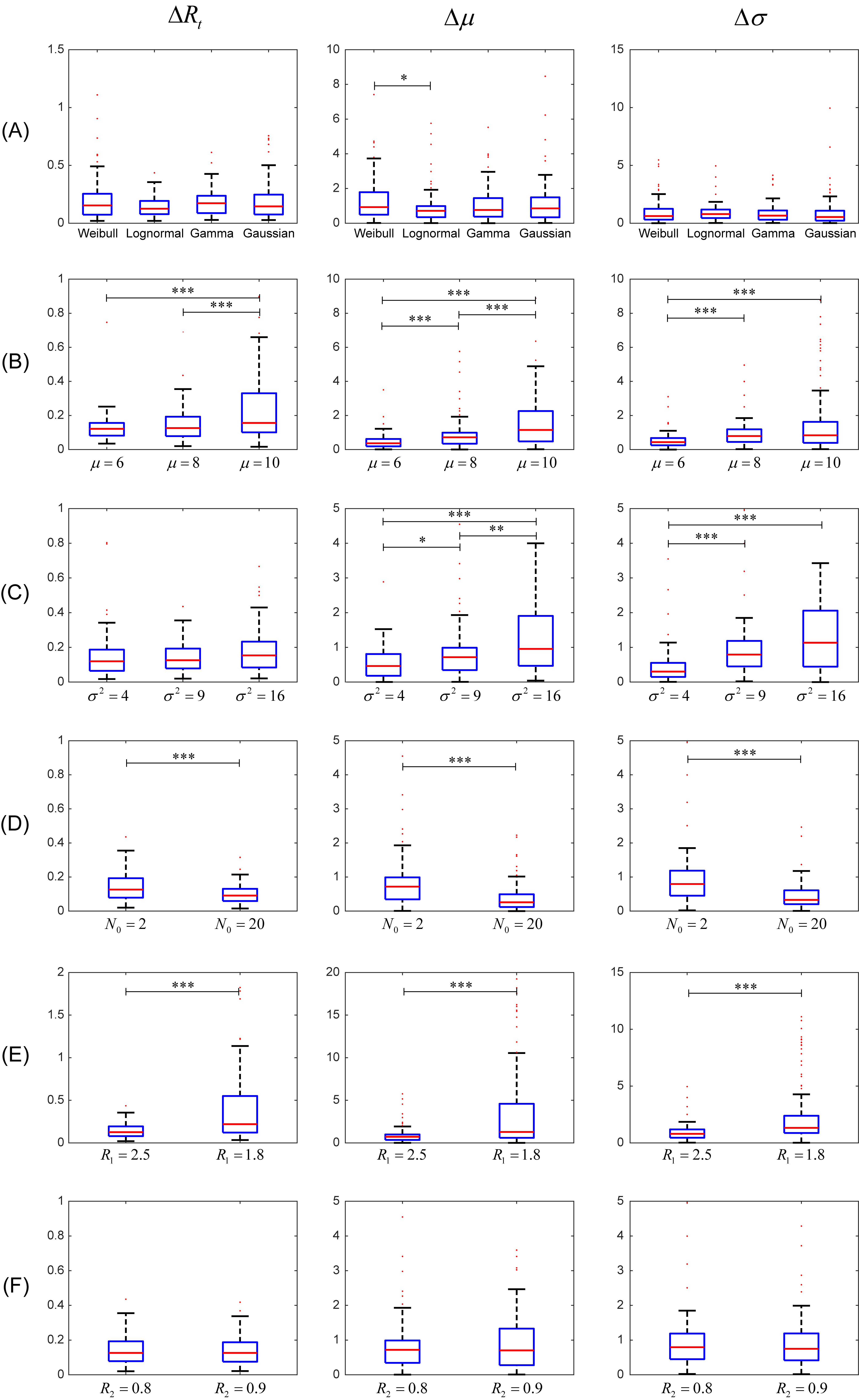


**S3 Fig**. The performance of our method after varying different parameters. The left, middle and right panels present the results for $\Delta R_{t}$, $\Delta\mu$, and $\Delta\sigma$, respectively. (A) Serial interval distribution. (B) Mean value of the serial interval. (C) Standard deviation of the serial interval. (D) The number of initial cases. (E) The epidemic severity R_1_. (F) The effectiveness of control measures R_2_. Simulations were performed based on the assumptions that the number of initial cases was 2, the serial interval exhibited a lognormal distribution with a mean and variance of 8 and 9, respectively, and a constant R before (R_1_=2.5) and after (R_2_=0.9) a control measure on day 40. When we varied a parameter to study its effects, the others were kept unchanged. *: P<0.05, **: P<0.01, and ***: P<0.001.
