# Supplementary material for: A new method for the joint estimation of instantaneous reproductive number and serial interval during epidemics
Source: PLoS Comput Biol. 2023 Mar 31;19(3):e1011021. doi: 10.1371/journal.pcbi.1011021 (PMC10096265; doi:10.1371/journal.pcbi.1011021)
Supplement: S4 Fig — (A) Distribution of serial interval over time. (B) Daily incidence generated by varying the serial interval. (C) Estimates of instantaneous reproductive number. (D) Estimates of serial interval. The red dashed curve denotes the true serial interval distribution averaged over time. (E) ΔRt. (F) Δμ is the absolute error between the true mean serial interval averaged over time and our estimates. (G) Δσ is the absolute error between the true standard deviation of the serial interval averaged over time and our estimates. A total of 100 trials were conducted to investigate the performance of our method by varying mean serial interval (from 8 to 3) and fixed mean serial interval (8). Simulations were performed based on the assumptions that the number of initial cases was 2, the serial interval exhibited a lognormal distribution with a variance of 9, and a constant R before (R1 = 2.5) and after (R2 = 0.9) a control measure on day 40. *: P<0.05, and ***: P<0.001. (DOCX) [file pcbi.1011021.s008.docx]

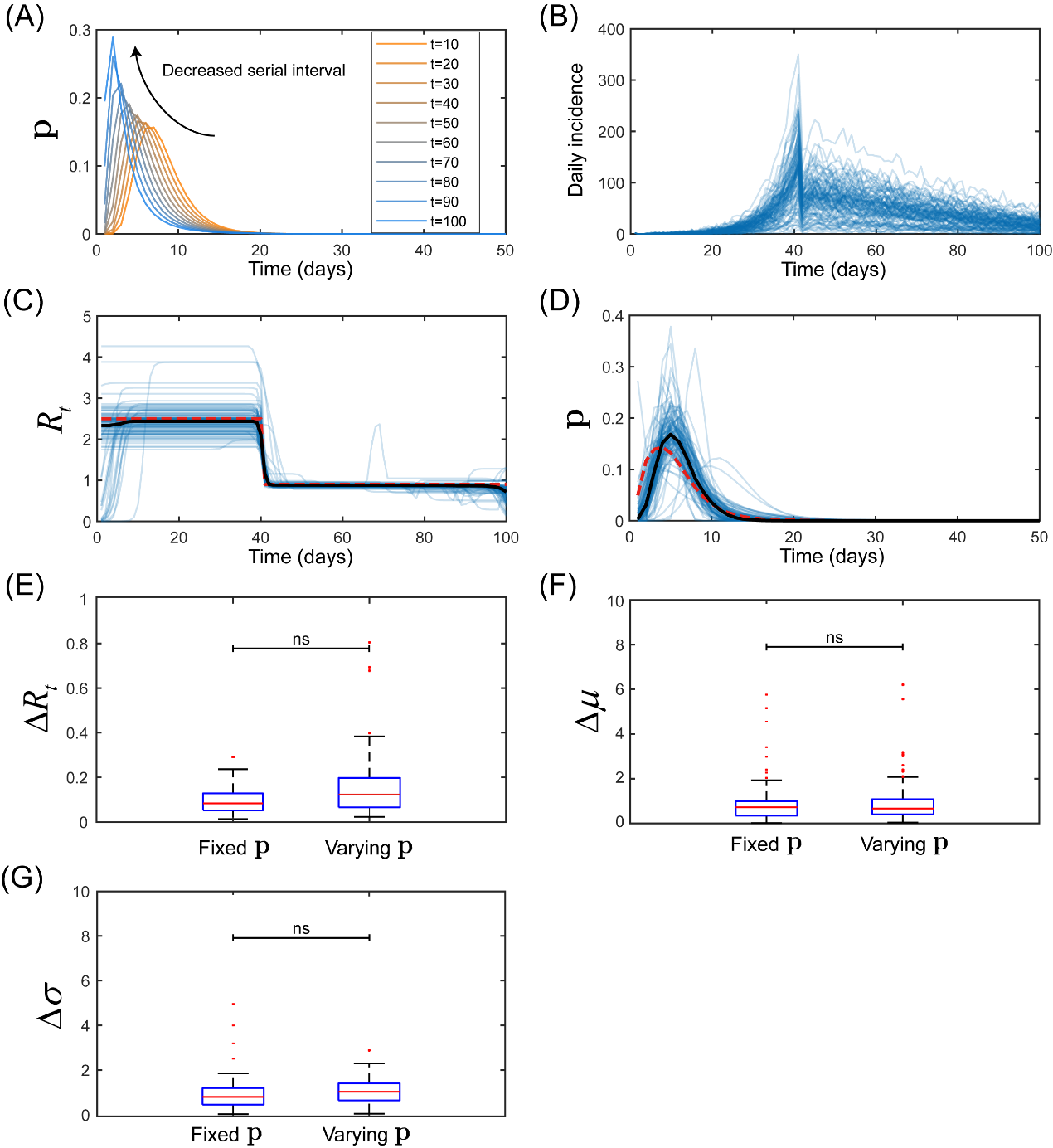


**S4 Fig**. Effects of changing the serial interval on the results. (A) Distribution of serial interval over time. (B) Daily incidence generated by varying the serial interval. (C) Estimates of instantaneous reproductive number. (D) Estimates of serial interval. The red dashed curve denotes the true serial interval distribution averaged over time. (E) $\Delta R_{t}$. (F) $\Delta\mu$ is the absolute error between the true mean serial interval averaged over time and our estimates. (G) $\Delta\sigma$ is the absolute error between the true standard deviation of the serial interval averaged over time and our estimates. A total of 100 trials were conducted to investigate the performance of our method by varying mean serial interval (from 8 to 3) and fixed mean serial interval (8). Simulations were performed based on the assumptions that the number of initial cases was 2, the serial interval exhibited a lognormal distribution with a variance of 9, and a constant R before (R_1_=2.5) and after (R_2_=0.9) a control measure on day 40. *: P<0.05, and ***: P<0.001.
