# Supplementary material for: A new method for the joint estimation of instantaneous reproductive number and serial interval during epidemics
Source: PLoS Comput Biol. 2023 Mar 31;19(3):e1011021. doi: 10.1371/journal.pcbi.1011021 (PMC10096265; doi:10.1371/journal.pcbi.1011021)
Supplement: S5 Fig — A total of 100 trials were conducted to investigate the performance of our method by varying the time length (from 40 to 200). Simulations were performed based on the assumptions that the number of initial cases was 2, the serial interval exhibited a lognormal distribution with a mean and variance of 8 and 9, respectively, and it was split into four stages. The left, middle and right panels are the estimates of instantaneous reproductive number, the estimates of the serial interval and the results of the three indices, respectively. The blue lines show the estimates, and the dark lines denote their mean values after 100 simulations. Additionally, the gray lines denote predictions and dashed dark lines represent their mean values after 100 simulations. The red dashed lines represent the ground truth. *: P<0.05, **: P<0.01, and ***: P<0.001. (DOCX) [file pcbi.1011021.s009.docx]

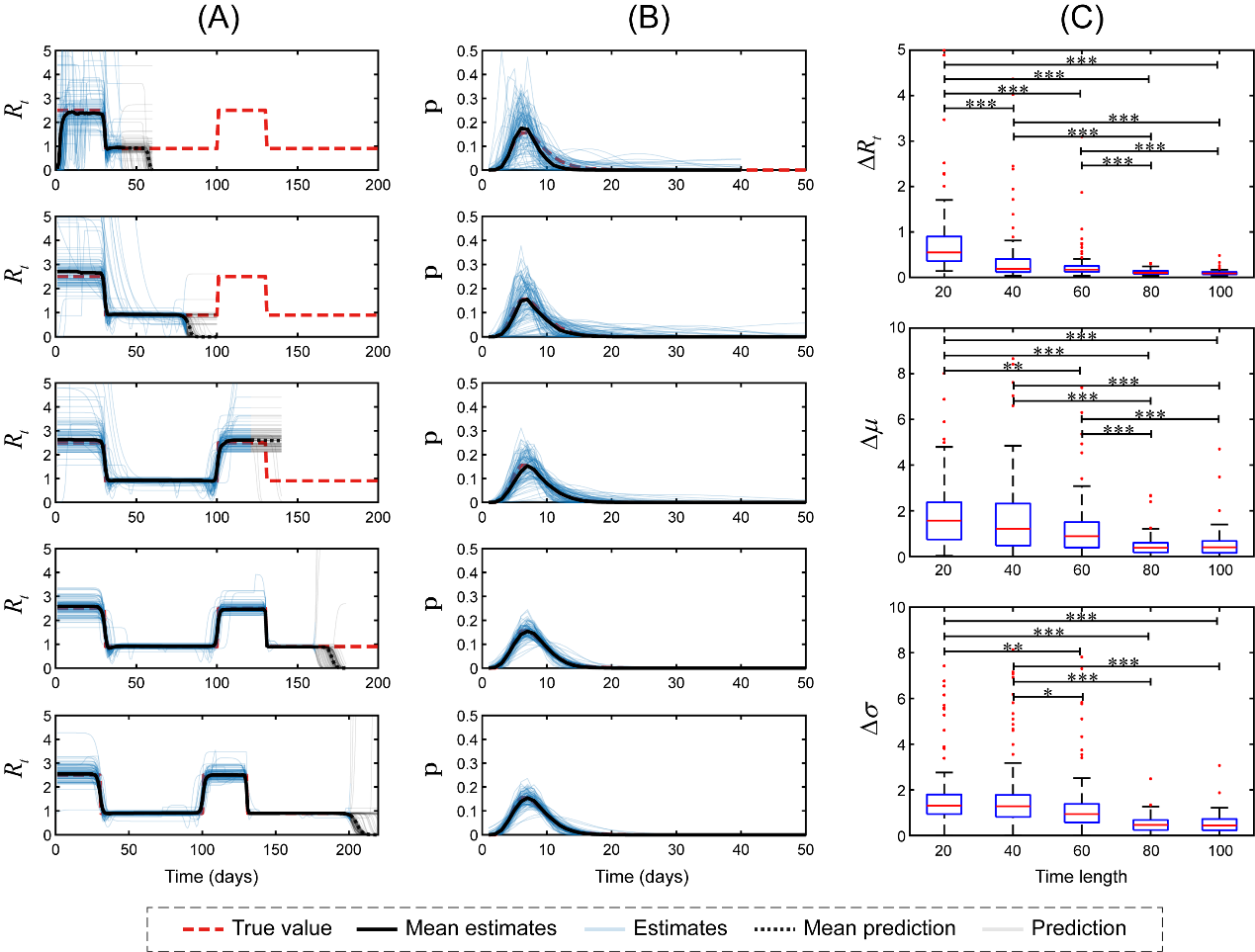


**S5 Fig**. The effects of time length on the results. A total of 100 trials were conducted to investigate the performance of our method by varying the time length (from 40 to 200). Simulations were performed based on the assumptions that the number of initial cases was 2, the serial interval exhibited a lognormal distribution with a mean and variance of 8 and 9, respectively, and it was split into four stages. The left, middle and right panels are the estimates of instantaneous reproductive number, the estimates of the serial interval and the results of the three indices, respectively. The blue lines show the estimates, and the dark lines denote their mean values after 100 simulations. Additionally, the gray lines denote predictions and dashed dark lines represent their mean values after 100 simulations. The red dashed lines represent the ground truth. *: P<0.05, **: P<0.01, and ***: P<0.001.
